# Structure of the Fibrillin-1 N-Terminal Domains Suggests that Heparan Sulfate Regulates the Early Stages of Microfibril Assembly

**DOI:** 10.1016/j.str.2013.08.004

**Published:** 2013-10-08

**Authors:** David A. Yadin, Ian B. Robertson, Joanne McNaught-Davis, Paul Evans, David Stoddart, Penny A. Handford, Sacha A. Jensen, Christina Redfield

**Affiliations:** 1 Department of Biochemistry, University of Oxford, South Parks Road, Oxford OX1 3QU, UK

## Abstract

The human extracellular matrix glycoprotein fibrillin-1 is the primary component of the 10- to 12-nm-diameter microfibrils, which perform key structural and regulatory roles in connective tissues. Relatively little is known about the molecular mechanisms of fibrillin assembly into microfibrils. Studies using recombinant fibrillin fragments indicate that an interaction between the N- and C-terminal regions drives head-to-tail assembly. Here, we present the structure of a fibrillin N-terminal fragment comprising the fibrillin unique N-terminal (FUN) and the first three epidermal growth factor (EGF)-like domains (FUN-EGF3). Two rod-like domain pairs are separated by a short, flexible linker between the EGF1 and EGF2 domains. We also show that the binding site for the C-terminal region spans multiple domains and overlaps with a heparin interaction site. These data suggest that heparan sulfate may sequester fibrillin at the cell surface via FUN-EGF3 prior to aggregation of the C terminus, thereby regulating microfibril assembly.

## Highlights

• The fibrillin unique N-terminal (FUN) domain adopts a novel fold
• The binding site for the fibrillin C terminus spans multiple domains
• The heparin interaction site overlaps with the C-terminal binding region
• Detailed molecular insights into an interaction between fibrillin molecules

## Teaser

Fibrillin microfibrils are key structural and regulatory components of connective tissue, but their assembly is poorly understood. Yadin et al. report a structure of the fibrillin N-terminal domains and propose that heparan sulphate regulates end-to-end assembly.

## Introduction

Fibrillins are the primary constituents of the 10- to 12-nm-diameter microfibrils in the extracellular matrix (ECM) of many elastic and non-elastic connective tissues. In elastic tissues such as the lamellae of blood vessels, the skin, and the lungs, they are present in elastic fibers in which they form a scaffold around an amorphous elastin polymer (Sakai et al., 1986). Fibrillin microfibrils are also found in elastin-free assemblies in nonelastic tissues such as the ciliary zonules of the eye and the kidney glomerulus. In addition to performing structural roles, fibrillin microfibrils contribute to the functional regulation of the ECM. They interact with cells via integrins (Pfaff et al., 1996) and sequester growth factors, notably latent transforming growth factor-β (TGF-β), through interactions of fibrillin with the latent TGF-β-binding proteins (LTBPs) (Ono et al., 2009) and the prodomains of several bone morphogenetic proteins (BMPs) (Sengle et al., 2008).

The importance of fibrillin microfibrils is highlighted by the spectrum of acquired and inherited connective tissue disorders associated with elastic fibers. For example, defects have been identified in patients with pulmonary emphysema, aneurysms, and pelvic organ prolapse (Yanagisawa and Davis, 2010). Marfan syndrome (MFS) and congenital contractural arachnodactyly were the first diseases to be linked to the human *FBN1* and *FBN2* genes, respectively, which encode the fibrillin-1 and fibrillin-2 isoforms (Lee et al., 1991). More recently, a number of so-called fibrillinopathies have been identified and characterized, including stiff skin syndrome (Loeys et al., 2010), Weill-Marchesani syndrome (Faivre et al., 2003), and the acromelic dysplasias (Le Goff et al., 2011).

**Figure 1:**
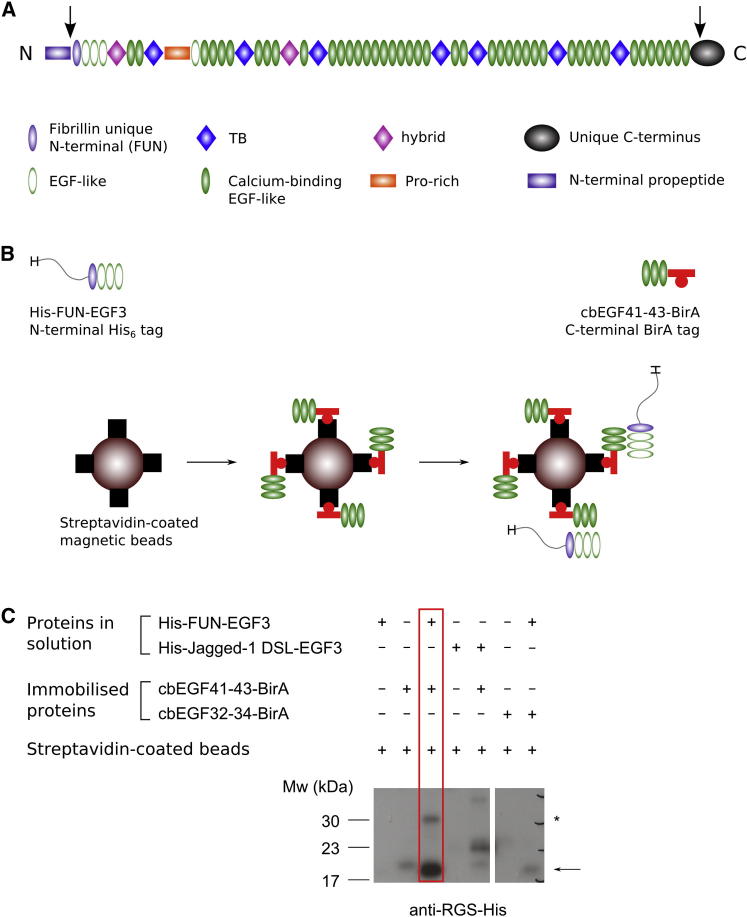
Fibrillin Domain Organization and N-C-Terminal Interaction (A) Domain structure of human fibrillin-1. Arrows indicate furin cleavage sites near the N- and C-termini: immediately preceding the fibrillin unique N-terminal domain and in the unique C-terminal region. (B) Pull-down assay: cbEGF41-43 was immobilized on streptavidin-coated magnetic beads and used to pull down His-tagged FUN-EGF3 from solution. (C) Binding was assessed by western blotting with an anti-RGS-His antibody. A separate blot with streptavidin-HRP confirmed the presence of immobilized proteins (Figure S1). The strong band at ∼17 kDa (arrow) in the boxed lane demonstrates the interaction between FUN-EGF3 and cbEGF41-43; a minor species at ∼30 kDa (asterisk) is occasionally observed. Binding of the negative control fragments fibrillin cbEGF32-34 (immobilized) and Jagged-1 DSL-EGF3 (soluble) to FUN-EGF3 and cbEGF41-43, respectively, was much weaker than that observed for the true interaction partners.

Fibrillins have a modular organization that is conserved from jellyfish to humans (Robertson et al., 2011). Their structures are dominated by calcium-binding epidermal growth factor-like (cbEGF) domains, of which there are 43 in human fibrillin-1 (Figure 1A). Arrays of cbEGF domains are interrupted by TGF-β-binding protein-like (TB) and hybrid (hyb) domains. In addition, there are unique N- and C-terminal regions that are processed by the protease furin (Kettle et al., 2000; Lönnqvist et al., 1998; Reinhardt et al., 2000). High-resolution structures of the major domain types have been determined with the use of nuclear magnetic resonance (NMR) spectroscopy and X-ray crystallography (Downing et al., 1996; Jensen et al., 2009; Yuan et al., 1997). In addition, fibrillin fragment structures show the architecture of the interdomain interfaces, which is important for understanding the conformation of the full-length molecule. There are presently no high-resolution structures of the N- and C-terminal regions, including the fibrillin unique N-terminal (FUN) domain, the unique C-terminal region, and the C-terminal propeptide.

Although there is a large body of work on intact microfibrils, isolated fibrillin molecules, and recombinant fragments, many molecular details regarding the assembly process and structural organization of microfibrils are still lacking. Microfibrils extracted from tissue have a periodic beaded filament structure, with an average of ∼56 nm repeat distance between the beads (Keene et al., 1991; Kielty et al., 1991). Because purified fibrillin molecules have a length of ∼150 nm (Sakai et al., 1991), two different models have been proposed to explain the observed periodicity of the beads. In the first model, fibrillin molecules adopt a linear conformation and are staggered in microfibrils (Kuo et al., 2007; Lee et al., 2004), whereas in the second, fibrillin monomers are folded (Baldock et al., 2006).

A common feature of all models of microfibril structure is the colocalization of fibrillin N- and C-terminal regions. This was first shown by labeling microfibrils with antibodies raised against recombinant fibrillin fragments (Reinhardt et al., 1996). Subsequently, an interaction between N- and C-terminal fibrillin fragments was demonstrated in vitro (Lin et al., 2002; Marson et al., 2005). The minimal interacting regions of human fibrillin-1 were later localized to the FUN and three non-calcium-binding EGF-like domains (FUN-EGF3) at the N-terminus (El-Hallous et al., 2007) and last three cbEGF domains (cbEGF41-43) at the C terminus (Hubmacher et al., 2008). Multimerization of the C terminus into bead-like structures was also found to enhance its binding to the N-terminus (Hubmacher et al., 2008). It has been proposed that the N-C-terminal interaction is an important step in the microfibril assembly pathway, mediating end-to-end assembly of fibrillin monomers. The N-terminal region of fibrillin also interacts with a multitude of other ECM molecules, including heparan sulfate (HS), which may regulate microfibril assembly (Cain et al., 2005; Tiedemann et al., 2001). In addition, the FUN-EGF3 region forms part of the binding sites for two BMP prodomains (Sengle et al., 2008; Sengle et al., 2011) and the LTBPs (Ono et al., 2009).

Here, we present the solution structure of the FUN-EGF3 region of human fibrillin-1. We show that it comprises two domain pairs connected by a flexible linker, and reveal the structure of the FUN domain. Furthermore, we demonstrate that multiple domains in FUN-EGF3 contribute to its binding to the cbEGF41-43 region. Using structure-informed mutagenesis, we show that a loop in the FUN domain and the flexible EGF1-EGF2 linker form part of the binding site, which overlaps with a heparin interaction site. Our data provide important insights into interactions involved in fibrillin microfibril assembly.

## Results

### Interaction of N- and C-Terminal Fibrillin-1 Fragments

An interaction between recombinant fragments corresponding to the FUN-EGF3 (residues R45–E178) and cbEGF41-43 (residues D2567–V2687) regions of human fibrillin-1 was confirmed using a pull-down assay prior to structure determination of FUN-EGF3. These fragments were produced using an established bacterial expression and in vitro refolding system (Knott et al., 1996). Low solubility of the cbEGF41-43 fragment at pH 7.4 necessitated its immobilization on magnetic beads using the streptavidin-biotin interaction. Immobilized, site-specifically biotinylated cbEGF41-43 with a C-terminal BirA tag was used to pull down FUN-EGF3 from solution (Figures 1B and 1C). Binding specificity was demonstrated using unrelated control fragments with sizes and structures similar to those of FUN-EGF3 and cbEGF41-43: the Delta-Serrate-Lag2 (DSL)-EGF3 fragment of human Jagged-1 (Cordle et al., 2008a) in place of FUN-EGF3, and the human fibrillin-1 cbEGF32-34 fragment instead of cbEGF41-43. The observed binding to cbEGF41-43 indicated that the refolded FUN-EGF3 fragment had native-like properties.

### Structure Determination of FUN-EGF3 and Analysis of Dynamics

**Figure 2:**
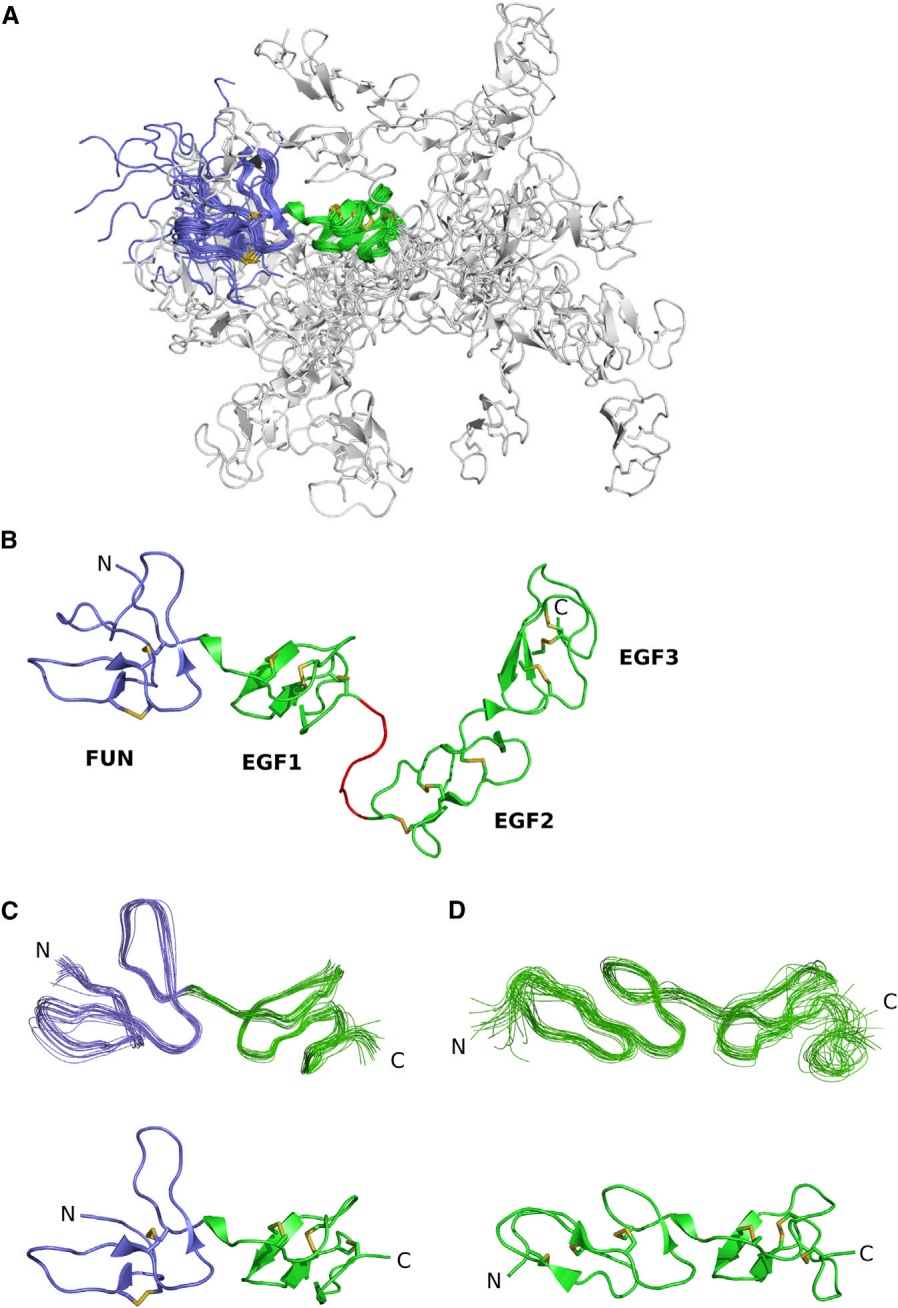
Final Structure Ensemble of FUN-EGF3 (A) Twenty-structure ensemble of FUN-EGF3, with structures aligned to the FUN-EGF1 region (cartoon representation). Disulphides are shown as sticks. The FUN and EGF1 domains are colored lilac and green, respectively, and the EGF2–EGF3 region is colored white. (B) Lowest-energy structure from the FUN-EGF3 ensemble, with the EGF1-EGF2 linker sequence (G112–H118) in red. (C and D) Backbone of the (C) FUN-EGF1 region, showing residues 52–112, and (D) EGF2–EGF3 region, showing residues 118–178. Energy-minimized average structures are shown below the aligned ensembles.

NMR spectroscopy was used to solve the structure of FUN-EGF3. The structure was determined by simulated annealing from an extended template using 2,599 nuclear Overhauser effect (NOE) and 137 ϕ and ψ torsion angle restraints (Table 1). Structures were refined using hydrogen bond restraints and residual dipolar couplings (RDCs). There were many NOEs between residues in the FUN and EGF1 domains, as well as between the EGF2 and EGF3 domains. However, no NOEs between the FUN-EGF1 and EGF2-EGF3 domain pairs were observed, which was reflected by the variability of their relative orientation in the 20-structure ensemble (Figure 2A). This was due to an unstructured seven-residue linker sequence between the EGF1 and EGF2 domains (residues G112–H118; Figure 2B). The N-terminal segment of the FUN domain (residues R45–A52) was similarly poorly defined. The disordered character of these regions was previously predicted from chemical shifts (Yadin et al., 2012). Therefore, separate alignment tensors for the RDC restraints were used for FUN-EGF1 and EGF2-EGF3 in refinement; average structures for the two units were calculated separately (Figures 2C and 2D).

**Figure 3:**
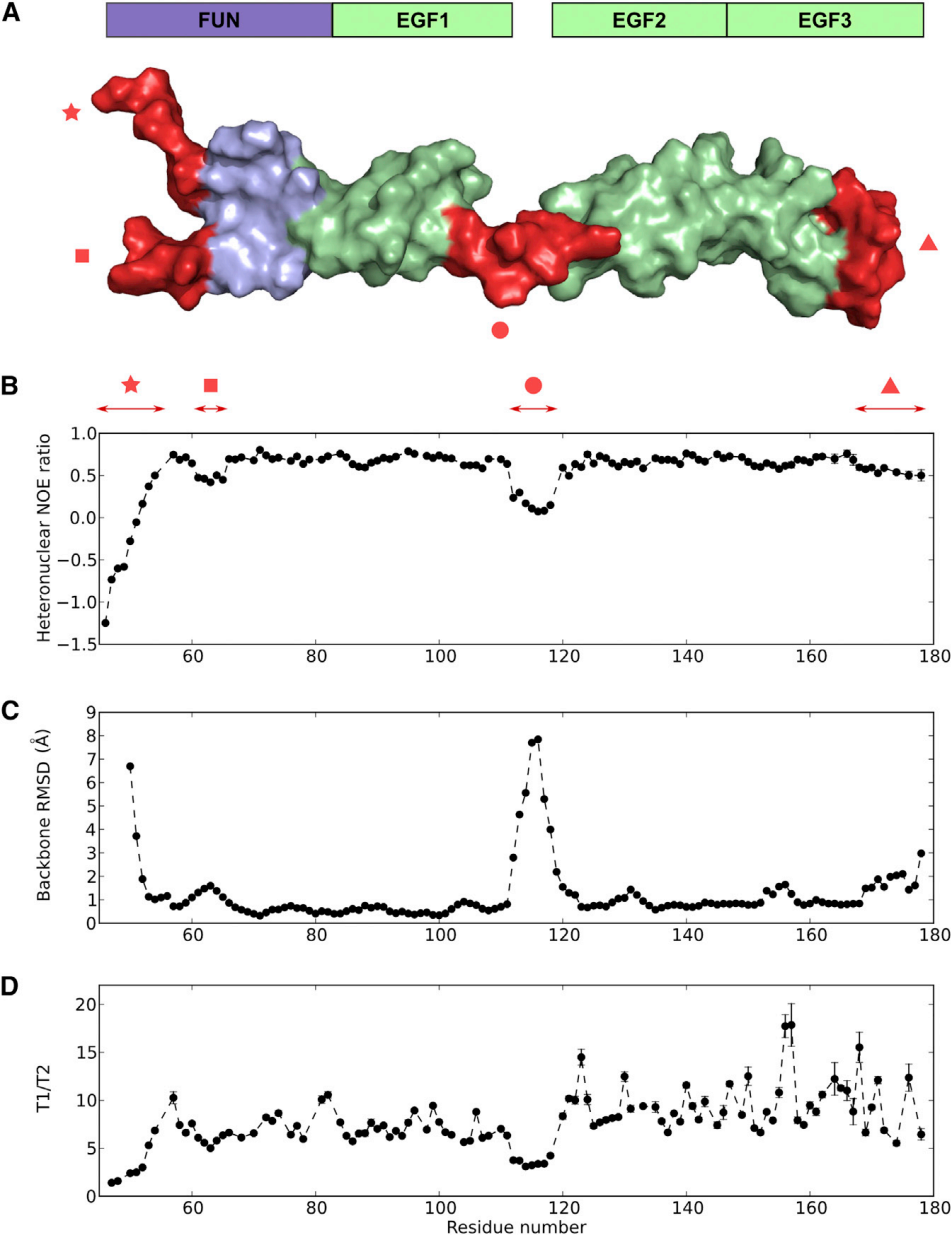
Dynamics and Structure Precision of FUN-EGF3 (A) Surface representation of a member of the FUN-EGF3 structure ensemble, with dynamic regions highlighted in red: N terminus (R45–A52; star), FUN domain loop (S61–A66; square), EGF1-EGF2 linker (G112–H118; circle), and C-terminal loop of EGF3 (T169–E178; triangle). (B) {^1^H}-^15^N heteronuclear NOE ratios plotted as a function of residue number. Dynamic regions highlighted in (A) are indicated by shapes. Asn/Gln side-chain data are presented in Table S1. (C) Backbone rmsd for each residue from the energy-minimized average structures of FUN-EGF1 (up to S115) and EGF2-EGF3 (from I116). N-terminal residues with rmsd > 10 Å are omitted for clarity. (D) T_1_/T_2_ values plotted against residue number. Errors in the {^1^H}-^15^N heteronuclear NOE ratios (B) and T_1_/T_2_ (D) were estimated using 500 Monte Carlo simulations, with the baseline noise as a measure of peak height error, as described previously (Bruylants and Redfield, 2009).

A study of the backbone dynamics of FUN-EGF3 confirmed the presence of the flexible EGF1-EGF2 linker and highlighted other dynamic regions (Figure 3A). Residues in the linker had lower heteronuclear NOE ratios compared with residues in structured regions, indicating increased mobility on the picosecond-to-nanosecond timescale (Figure 3B). The experiment also showed flexibility at the N terminus, as well as a loop in the FUN domain (S61–A65) and the C-terminal portion of the EGF3 domain (C168–E176). The profile of heteronuclear NOE ratios was consistent with the backbone root-mean-square deviation (rmsd) values for members of the structure ensemble (Figure 3C). In addition, the variability of the T_1_/T_2_ ratios across the backbone indicated that many residues, particularly those in the EGF3 domain, exhibited motion on slower timescales (Figure 3D). In full-length fibrillin, EGF3 is likely to form an interface with the hyb1 domain. Several putative packing residues have different chemical shifts in a four-domain fragment comprising domains EGF2–cbEGF1 (Robertson et al., 2013). Overall, these results supported the structure and identified considerable internal dynamics on both fast and slow timescales.

### Domain Structures

**Figure 4:**
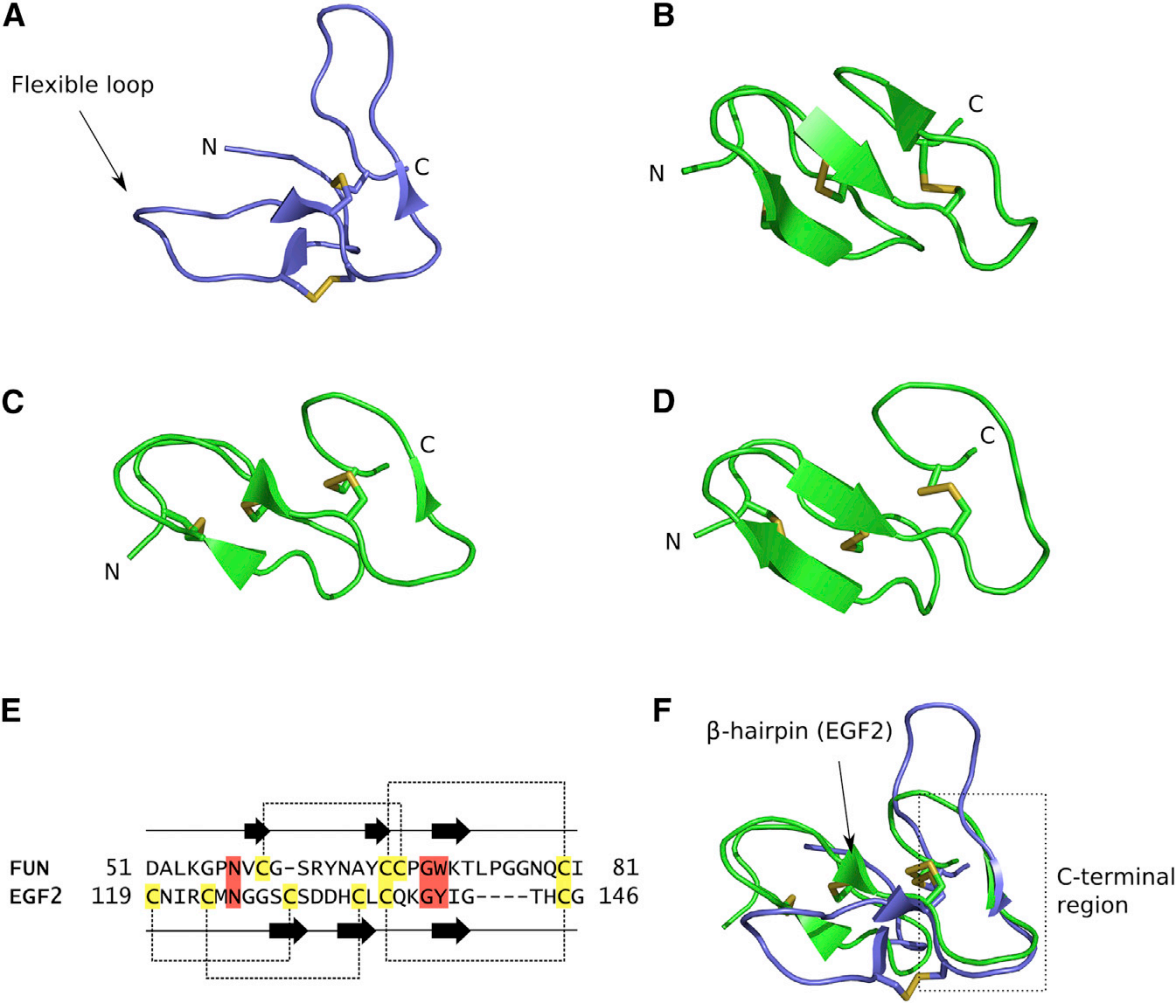
Domain Structures in the FUN-EGF3 Fragment (A) The FUN domain comprises two loops (one of which is flexible) linked by two disulphide bonds in a 1-3, 2-4 pattern (C59-C68 and C67-C80). (B) The EGF1 domain has a canonical 1-3, 2-4, 5-6 disulphide bond arrangement and a three-stranded antiparallel β sheet. (C and D) Structure of the EGF2 (C) and EGF3 (D) domains, which contain a β-hairpin, is shown. (E) Comparison of the FUN and EGF2 primary structure. Sequences were aligned manually on the basis of structural homology. Arrows represent stretches of β-sheet-like secondary structure and dotted lines indicate disulphide bonds. (F) Backbone of the EGF2 domain (green) superimposed on the FUN domain (lilac), illustrating the similarity of their C-terminal portions (dashed box). The β-hairpin in EGF2 is indicated.

The FUN-EGF3 structure shows the fold of the FUN domain, which was previously unknown (Figure 4A). With the exception of the unstructured N-terminal residues (R45–A52), the FUN domain adopts a compact conformation, but with little regular secondary structure. It comprises two loops connected by disulphide bonds, with an N-terminal segment that packs in between. The disulphide bond pairings of C59-C68 and C67-C80 (C1-C3 and C2-C4) were initially determined dynamically in the simulated annealing calculations and were supported by NOEs between cysteine side-chain resonances. The three EGF-like domains in the FUN-EGF3 fragment have a canonical EGF-like fold, with a disulphide bond arrangement of C1-C3, C2-C4, and C5-C6 (Bork et al., 1996; Figures 4B–4D). They all contain a β-hairpin motif, but the EGF1 domain also has a third minor β strand. Despite little obvious sequence identity between the FUN and EGF-like domains (Figure 4E), their C-terminal segments are structurally similar (Figure 4F). Instead of the β-hairpin structure of the EGF-like domain, the FUN domain has a flexible loop. Unlike the EGF β-hairpin, the loop in the FUN domain is not disulphide bonded to the N-terminal segment of the domain. This may explain the differences in the dynamic behavior of the two domains and could have functional significance.

### Interdomain Interfaces

**Figure 5:**
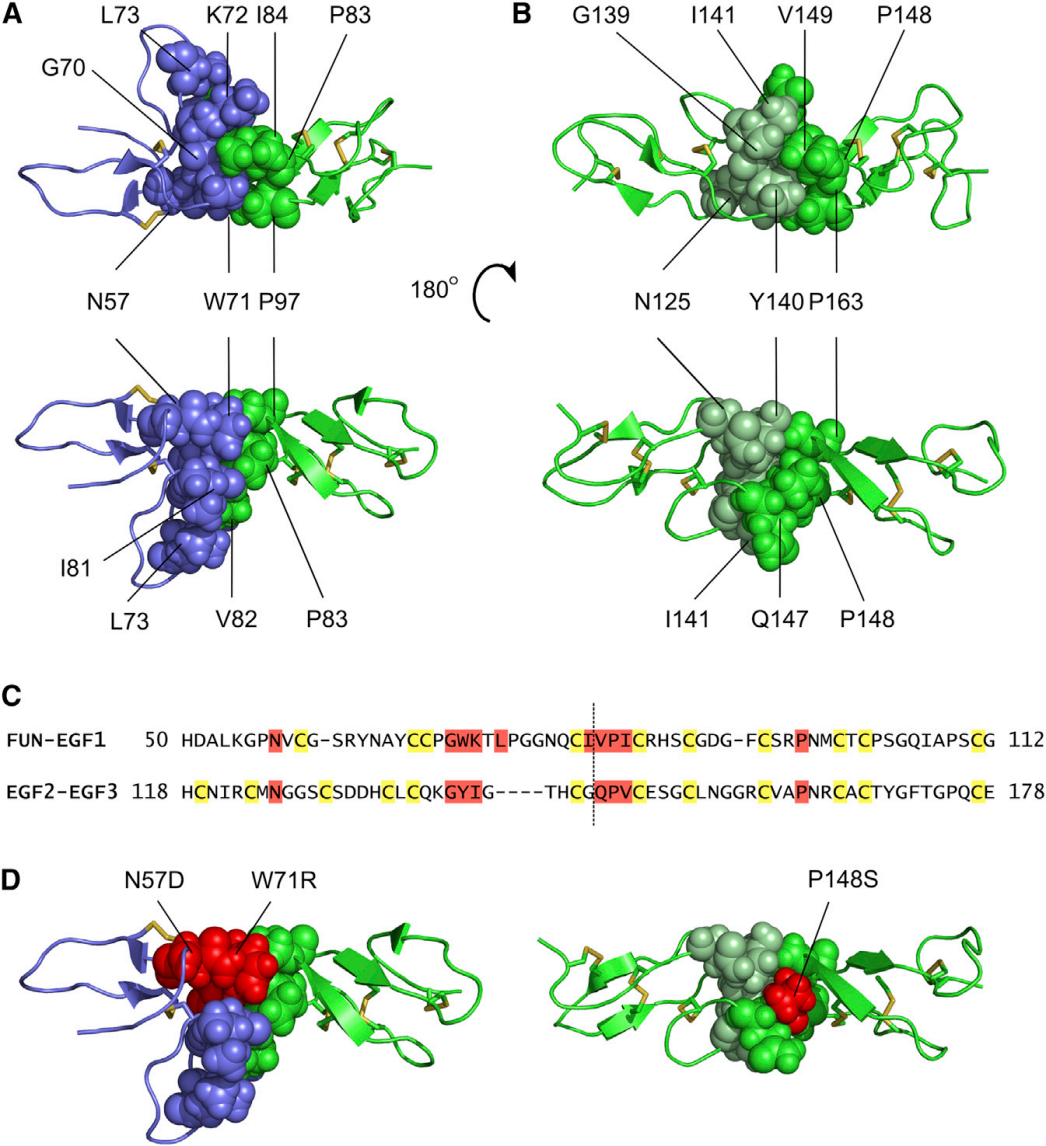
Interdomain Interfaces in FUN-EGF3 (A) Packing interactions between the FUN (lilac) and EGF1 (green) domains, showing two views of opposite faces. Residues involved in interdomain packing are shown as spheres and are colored according to domain. Packing residues were identified using a 4 Å interatomic distance cutoff as described previously (Jensen et al., 2009). (B) EGF2-EGF3 packing interactions. Residues from EGF2 and EGF3 involved in packing are colored light green and green, respectively. (C) Sequence alignment of the FUN-EGF1 and EGF2-EGF3 regions. Cysteines and packing residues are colored yellow and red, respectively. The dotted line delimits the domain boundaries. (D) Residues at interdomain interfaces that are substituted as a result of MFS-associated mutations (N57D and W71R in the FUN domain and P148S in EGF3). Further data for these mutations are given in Figure S2 and Table S2.

Interdomain interfaces within the FUN-EGF1 and EGF2-EGF3 domain pairs are well defined, but there is no observed interface between the EGF1 and EGF2 domains. Buried surface areas at the interfaces of the FUN-EGF1 and EGF2-EGF3 pairs, calculated from the energy-minimized average structures, were found to be 290 and 239 Å^2^, respectively. The areas buried by these interfaces are larger than those buried in a cbEGF-cbEGF pair, but smaller than those of TB-cbEGF and hyb-cbEGF interfaces: 184 Å^2^ for cbEGF32-cbEGF33 (Downing et al., 1996), 552 Å^2^ for TB4-cbEGF23 (Lee et al., 2004), and 670 Å^2^ for hyb2-cbEGF10 (Jensen et al., 2009). The FUN-EGF1 interface is formed by G70, W71, K72, L73, and I81 in the FUN domain, and V82, P83, I84, and P97 in the EGF1 domain (Figure 5A). Similarly, the EGF2-EGF3 interface is formed by G139, Y140, and I141 in the EGF2 domain, analogous to the G-W-K motif in the FUN domain, and the packing residues from EGF3 are Q147, P148, V149, and P163, similar to the EGF1 domain (Figure 5B). EGF2 does not have an analog of L73 in the FUN domain, accounting for the larger surface area buried by the FUN-EGF1 interface.

Aligning the sequences of the FUN-EGF1 and EGF2-EGF3 pairs highlights the similar arrangement of packing residues (Figure 5C). Residues at equivalent positions in the EGF1 and EGF2 domains are absent, explaining the lack of an interdomain interface. In addition, residues N57 and N125 from the FUN and EGF2 domains, respectively, pack against the aromatic rings of W71 and Y140, and are likely to contribute to the stability of the interdomain interfaces. The NH_2_ side-chain groups of these two residues are less mobile than surface-exposed side-chains (Table S1 available online), supporting their roles in interdomain packing.

Interestingly, three missense mutations found in patients with MFS result in substitutions of residues at the interdomain interfaces: N57D (Chung et al., 2009) and W71R (Sakai et al., 2006) in the FUN domain and P148S in the EGF3 domain (Figure 5D; Stheneur et al., 2009). These substitutions could disrupt interdomain interfaces, resulting in misfolding and intracellular retention, as was previously observed for several MFS mutations (Whiteman et al., 2007). Introduction of the N57D and P148S substitutions into the FUN-EGF3 fragment resulted in local misfolding of the FUN and EGF3 domains, respectively, whereas the W71R substitution caused global misfolding (Table S2; Figure S2). Furthermore, introduction of the N57D and W71R substitutions into a longer N-terminal fragment resulted in partial retention of the protein by fibroblast cells (Figure S2). This may suggest a pathogenic mechanism of functional haploinsufficiency for these mutations.

### Homologs of the FUN-EGF1 and EGF2-EGF3 Domain Pairs

**Figure 6:**
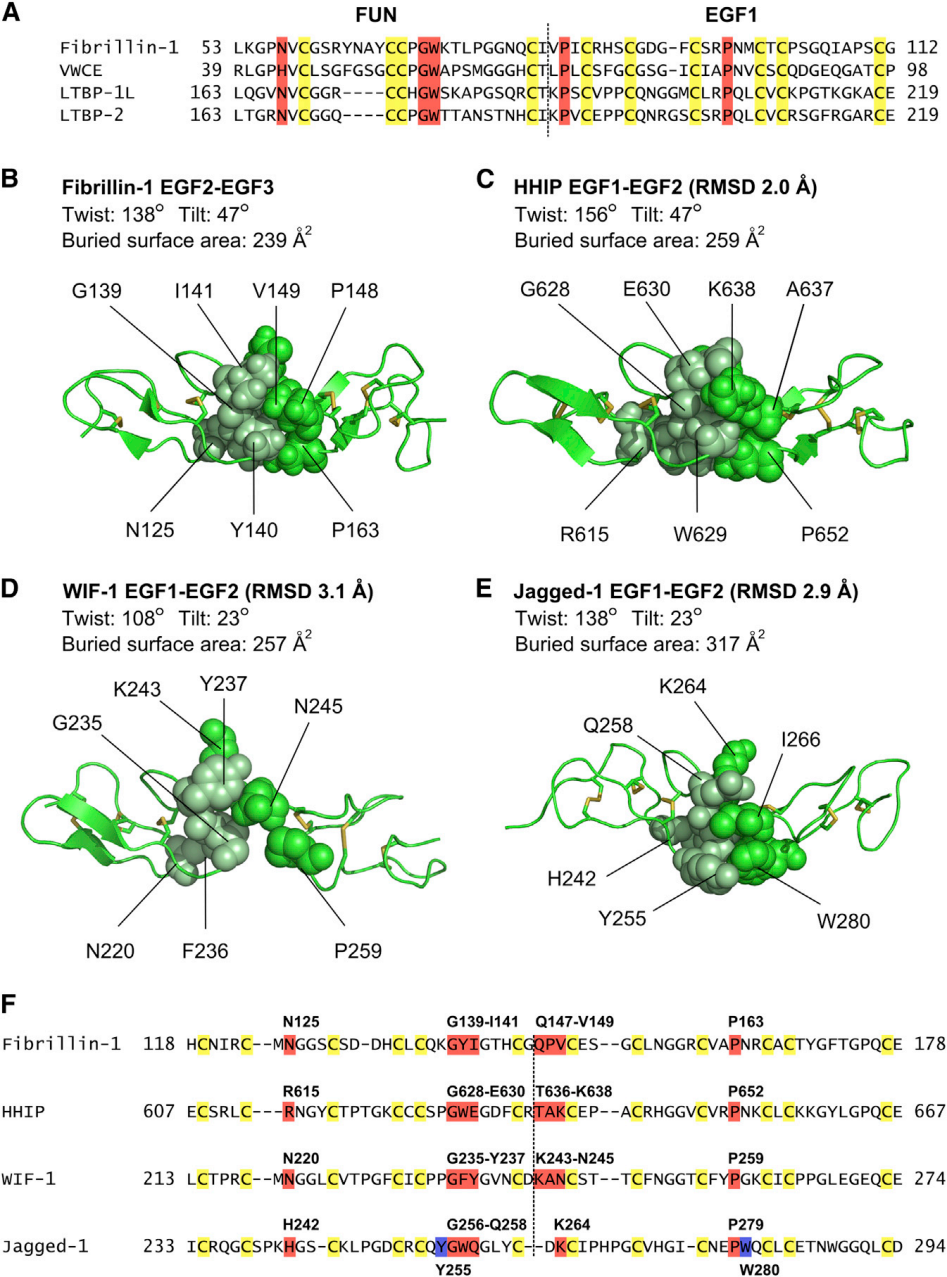
Homologs of the FUN-EGF1 and EGF2-EGF3 Domain Pairs (A) Sequence alignment of homologs of the FUN-EGF1 domain pair in other proteins (LTBP-1L, LTBP-2, and VWCE). The LTBPs lack the four-residue sequence preceding the C-C motif. (B–E) Packing residues in EGF-EGF domain pairs, also showing the interdomain twist and tilt angles. The backbone rmsd from the fibrillin-1 EGF2-EGF3 pair, calculated using the Superpose server (Maiti et al., 2004), is given for the three homologs. (B) Fibrillin-1 EGF2-EGF3. (C) Human hedgehog interacting protein (HHIP) EGF1-EGF2 (PDB ID 3H0B). (D) Wnt inhibitory factor-1 (WIF-1) EGF2-EGF3 (PDB ID 1IVO). (E) Human Jagged-1 EGF1-EGF2 (PDB ID 2V2J). (F) Sequence alignment of fibrillin-1, HHIP, WIF-1, and Jagged EGF-EGF domain pairs. Packing residues are colored red and indicated above the sequences. Additional packing residues in Jagged-1 are highlighted in blue.

The Dali server (Holm and Rosenström, 2010) did not find any structural homologs of the FUN-EGF1 domain pair, suggesting that the FUN domain has a novel fold. However, there are domain pairs with homologous sequences at the N termini of human LTBP-1L (long isoform) and LTBP-2 (Robertson et al., 2011), as well as in the liver-specific von Willebrand factor C and EGF domain-containing protein (VWCE/URG11) (Lian et al., 2006). Sequence alignments show an identical arrangement of cysteines and the presence of several of the key packing residues from fibrillin-1 FUN-EGF1 in LTBP-1L (Figure 6A). Strikingly, a conserved four-residue (^63^Y-N-A-Y^66^) motif in the flexible loop of the fibrillin-1 FUN domain is missing from the LTBPs.

There are several homologs of the EGF2-EGF3 domain pair with known structures: the EGF1-EGF2 pair in human hedgehog interacting protein (HHIP) (Bishop et al., 2009), EGF2-EGF3 in Wnt inhibitory factor-1 (WIF-1) (Malinauskas et al., 2011), and EGF1-EGF2 in human Jagged-1 (Cordle et al., 2008a). Like the fibrillin-1 EGF2-EGF3 pair, they all adopt a rod-like conformation, but there is variation in the twist and tilt angles and the buried surface area (Figures 6B–6E). The G-F/Y/W motif in the N-terminal EGF and the proline residue between C3 and C4 of the C-terminal EGF domain are found in all four domain pairs, but other packing residues differ (Figure 6F). The sequence variation presumably reflects the different functional roles of the EGF-domain-containing proteins.

### Sequence Conservation

**Figure 7:**
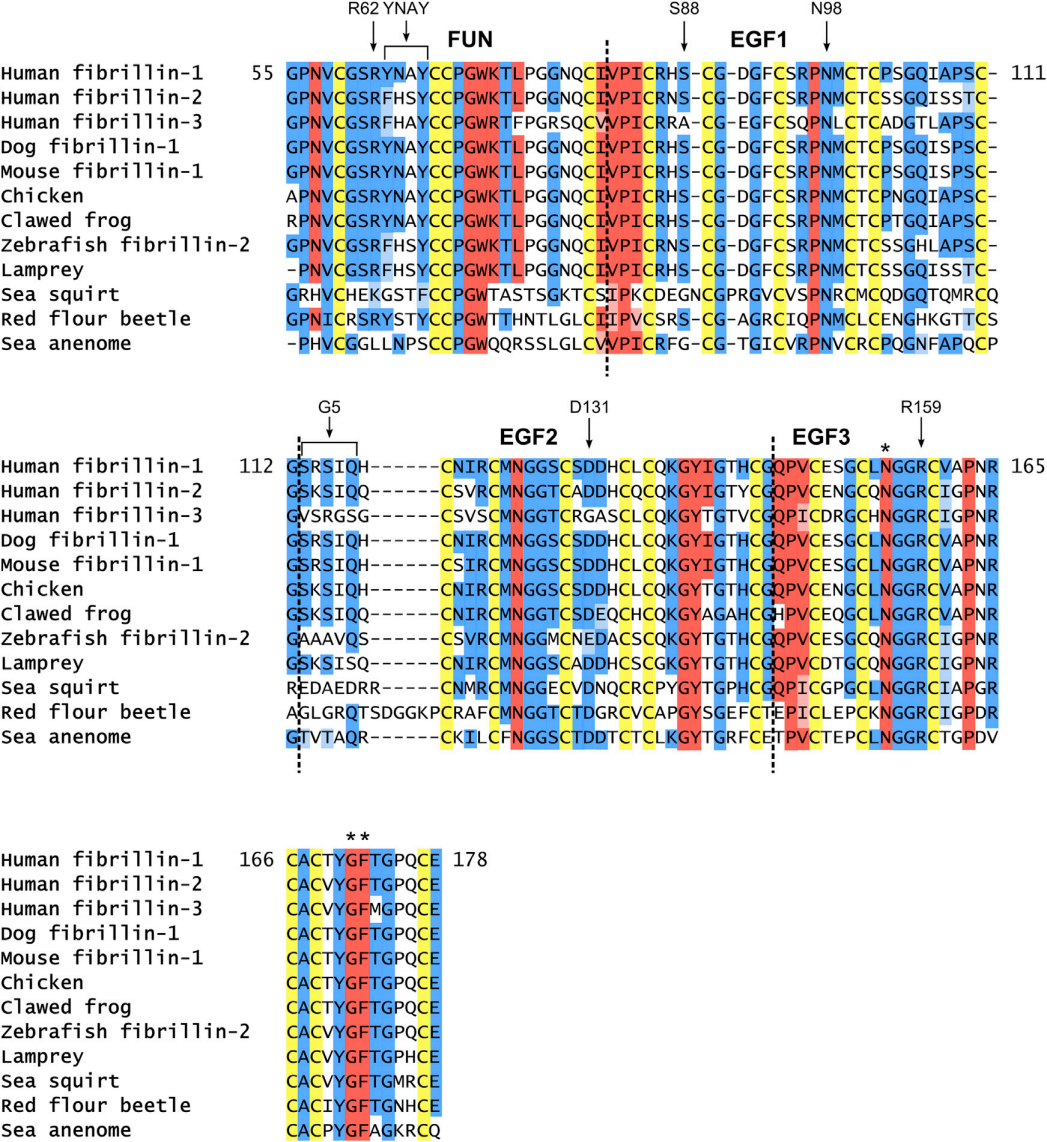
Sequence Alignments of the FUN-EGF3 Region Sequences of the FUN-EGF3 region from a range of species were obtained from NCBI and Ensembl databases (Table S3) and aligned using ClustalOmega. Residue numbers are given for human fibrillin-1. Cysteines and packing residues identified from the structure of fibrillin-1 FUN-EGF3 are colored yellow and red, respectively. Residues in the EGF3 domain that pack against the hyb1 domain have not yet been well defined, but N156 and the G171-F172 motif (indicated by ^∗^) are also highlighted in red, according to their similarity to packing residues at the FUN-EGF1 and EGF2-EGF3 interdomain interfaces and chemical-shift differences from the EGF2-cbEGF1 fragment (Robertson et al., 2013). Packing motifs are present in sequences from all species shown, but not every residue is fully conserved. Other conserved residues are colored blue; similar residues are shown in a lighter shade. Highlighted conserved residues are present in at least one of the three species lacking LTBPs, fibulins, and MAGPs (sea squirt, red flour beetle, and sea anemone) (Robertson et al., 2011). Residues targeted here by mutagenesis are indicated.

Sequence alignments of the FUN-EGF3 region from the three human fibrillin isoforms and a variety of fibrillin-containing species show a significant conservation across the evolutionary tree. The arrangement of cysteines is absolutely conserved, as are many of the packing residues in fibrillin-1 (Figure 7). By contrast, the flexible linker sequence between EGF1 and EGF2 is longer in several invertebrate species, particularly in the red flour beetle *Tribolium castaneum*. Within the structured domains of FUN-EGF3, there are a number of conserved residues that do not have an obviously structural role. Interpretation of sequence conservation in terms of function is not straightforward because of the large number of ECM components that may interact with the fibrillin N-terminal domains. However, fibrillin microfibrils are found in several lower metazoa, such as jellyfish (Reber-Müller et al., 1995), supporting the universal role of fibrillins as microfibril-forming proteins. By contrast, many potential interaction partners, including the LTBPs and microfibril-associated glycoproteins (MAGPs), are not found in all fibrillin-containing organisms (Robertson et al., 2011). Residues conserved from humans to lower metazoa may be implicated in the N-C-terminal interaction and thereby also microfibril assembly.

### Dissecting the Fibrillin N-C Interaction

**Figure 8:**
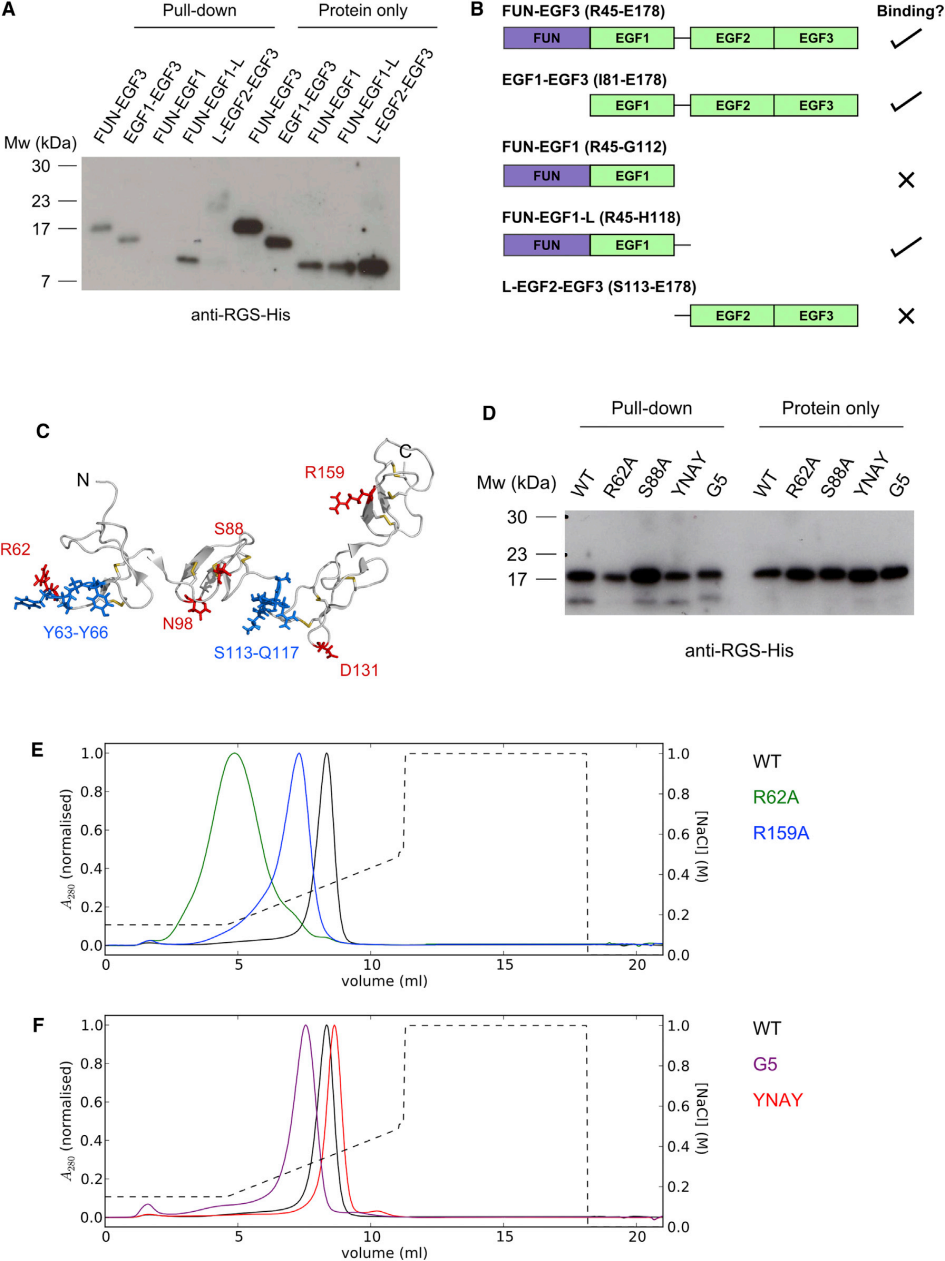
Mapping the Binding Sites for cbEGF41-43 and Heparin on FUN-EGF3 (A) Pull-down assay using immobilized cbEGF41-43 with various N-terminal fragments (FUN-EGF3, EGF1-EGF3, FUN-EGF1, FUN-EGF1-L [L denotes with the EGF1-EGF2 linker], and L-EGF2-EGF3). No significant binding was detected for FUN-EGF1 or L-EGF2-EGF3. See also Figure S1. (B) Summary of results for pull-downs of shorter fragments. (C) FUN-EGF3 structure showing residues targeted by site-directed mutagenesis. Five single residues were substituted with alanine (red), ^63^Y-N-A-Y^66^ in the FUN domain (blue) was deleted, and ^113^S-R-S-I-Q^117^ in the EGF1-EGF2 linker (blue) was replaced with five glycines. (D) Pull-down assay with FUN-EGF3 mutants. R62A, YNAY, and G5 all show reduced binding relative to wild-type (WT) and S88A (positive control) FUN-EGF3, although very similar quantities were added to the beads (“protein only”). Streptavidin-HRP blots confirmed the presence of cbEGF41-43 on the beads (Figure S1). Correct folding of the N-terminal fragments and the proteins containing mutations was shown by SDS-PAGE and NMR (Figure S3). (E) Elution profiles for binding of WT, R62A, and R159A FUN-EGF3 to a heparin column; A_280_ values are normalized to the maximum for each trace. Proteins were bound to the column and eluted on a NaCl gradient (dashed line). (F) Heparin elution profiles for YNAY and G5 FUN-EGF3.

Molecular details of the N-C interaction were investigated using information from the FUN-EGF3 sequence and structure. Attempts to use NMR to map the binding surface of cbEGF41-43 on FUN-EGF3 were not successful. Peak intensities decreased uniformly as cbEGF41-43 was added to ^15^N-labeled FUN-EGF3 (data not shown), which may be due to the low solubility of cbEGF41-43 noted above. Instead, a dissection approach using smaller N-terminal fragments was used in the pull-down assay with immobilized cbEGF41-43 (Figures 8A and 8B). The domain pairs FUN-EGF1 and EGF2-EGF3 (containing the EGF1-EGF2 linker sequence ^113^S-R-S-I-Q-H^118^, denoted L-EGF2-EGF3) did not show appreciable binding to cbEGF41-43. In contrast, the EGF1-EGF3 fragment did bind to cbEGF41-43. Addition of the EGF1-EGF2 linker sequence to the C terminus of FUN-EGF1 led to detectable binding, suggesting that EGF1 in combination with the linker plays an important role in the interaction.

Several FUN-EGF3 mutants were also tested in the pull-down assay. Five conserved residues (R62, S88, N98, D131, and R159) were individually substituted with alanine (Figure 8C). They were chosen on the basis of their evolutionary conservation (Figure 7) and surface exposure. None of these abolished the interaction with cbEGF41-43, but R62A FUN-EGF3 showed reduced binding compared with wild-type FUN-EGF3 (Figure 8D and data not shown). A mutant lacking the ^63^Y-N-A-Y^66^ motif in the FUN domain showed reduced binding to cbEGF41-43. Replacement of the ^113^S-R-S-I-Q^117^ linker motif with five glycines (G5) also resulted in reduced binding (Figure 8D). Together, the fragment and mutant results support a binding surface that spans at least FUN-EGF1 and the EGF1-EGF2 linker region in FUN-EGF3. EGF2 and EGF3 do not make a detectable contribution to high-affinity binding.

### Heparin Binding

An N-terminal fibrillin fragment encompassing the region from the FUN domain to the EGF4 domain (FUN-EGF4) binds to heparin (Cain et al., 2005; Tiedemann et al., 2001). Similarly, FUN-EGF3 bound to a heparin column and was eluted on a NaCl gradient (Figure 8E). Substitution of positively charged residues had variable effects on heparin binding. R62A FUN-EGF3 passed straight through the column, whereas R159A FUN-EGF3 eluted at a lower NaCl concentration than the wild-type protein. Deletion of the ^63^Y-N-A-Y^66^ motif only had a minor effect on heparin binding, and the G5 mutant (lacking R114) eluted earlier than the wild-type (Figure 8F). These results indicate that arginine residues, particularly R62 in the FUN domain, are involved in the binding of FUN-EGF3 to heparin.

## Discussion

Here, we present the structure of the FUN-EGF3 region of human fibrillin-1, showing the novel fold of the FUN domain and architecture of the interdomain interfaces. The structure provides insights into the organization of the fibrillin N-terminal domains, which is important for understanding the overall shape of fibrillin in microfibrils. In addition, structure-informed mutagenesis of FUN-EGF3 enabled us to characterize its interaction with the three C-terminal cbEGF domains of fibrillin, cbEGF41-43, and heparin.

This study adds to the set of high-resolution structures of fibrillin domain types and interdomain interfaces. Previous work elucidated the structures of the major domain types in fibrillin, i.e., the cbEGF (Downing et al., 1996), TB (Yuan et al., 1997), and hyb domains (Jensen et al., 2009). However, the remaining regions with unknown structures, such as the N terminus, are some of the most functionally important. A previous study of evolutionary conservation showed that the region encoded by exon 2 of the *FBN1* gene (P56–I81) is largely conserved, especially the spacing of cysteines and other residues shown here to be involved in interdomain interactions (Piha-Gossack et al., 2012). In the FUN-EGF3 structure, this region makes up most of the structured “core” of the FUN domain. Furthermore, residues L53–G55, encoded by exon 1, form part of the core—a feature that could not have been predicted from amino acid sequences. Although it is unique within fibrillin, the FUN domain has homologs in the human proteins LTBP-1L/2 and VWCE, which are also associated with a C-terminal EGF-like domain. Given the similarity of the C-terminal portions of the FUN and EGF2 domains, it is possible that the FUN-EGF pair evolved from an EGF-EGF domain pair.

**Figure 9:**
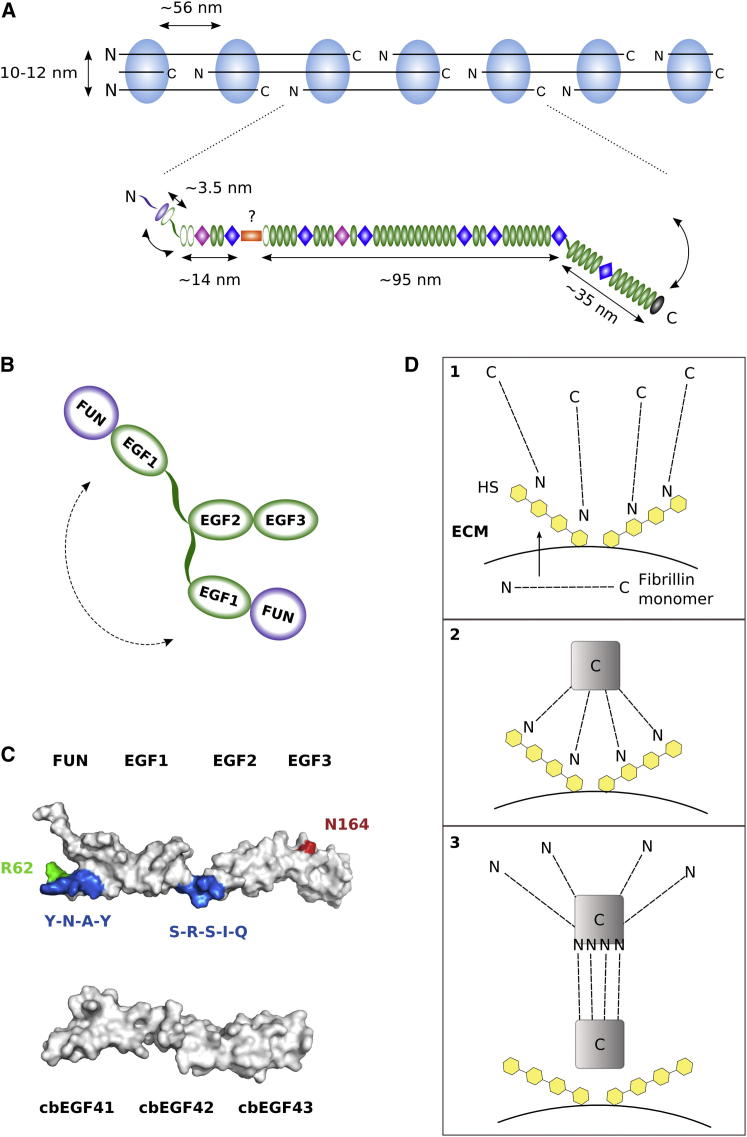
Implications of the FUN-EGF3 Structure for Microfibril Structure and Assembly (A) Fibrillin organization in microfibrils. Microfibrils extracted from tissue have a “beads-on-a-string” appearance; the diameter and interbead distances are indicated. An elongated arrangement of fibrillin monomers, each spanning two interbead regions of the microfibril, is shown according to the most recent version of the staggered model (Kuo et al., 2007). Approximate dimensions are given for three regions separated by the Pro-rich and TB6 domains using the following lengths: 2.7 nm, 2.3 nm, 2.0 nm, and 2.1 nm for cbEGF, TB, hyb, and non-calcium binding EGF-like domains, respectively. Flexibility of interdomain linkers is indicated by arrows. (B) The flexibility of the EGF1-EGF2 linker sequence means that the relative orientation of the FUN-EGF1 and EGF2-EGF3 pairs is variable, which may facilitate binding to cbEGF41-43. (C) Surface representation of a FUN-EGF3 structure from the ensemble, showing the regions involved in binding to cbEGF41-43, as well as N164 (LTBP-4 binding). Multiple domains of cbEGF41-43 (homology model based on cbEGF32-33 structure) span the interacting regions of FUN-EGF3. (D) Hypothetical model for HS regulation of the fibrillin N-C-terminal interaction. Secreted fibrillin monomers are sequestered on the cell surface by HS through binding to FUN-EGF3 (1). Oligomerization of the C-terminal domains (2) creates a high-affinity binding site that competes with HS for binding to FUN-EGF3 (3).

Studying the structure and dynamics of fibrillin is important for understanding microfibril organization. Previous high-resolution structures indicated that fibrillin monomers adopt linear conformations, consistent with staggered models of microfibril structure (Downing et al., 1996; Jensen et al., 2009; Lee et al., 2004). Likewise, the EGF2-EGF3 domain pair has a rod-like shape. However, the flexible linker between the EGF1 and EGF2 domains is strikingly different from most other regions of fibrillin. An earlier study showed that the linker between the TB6 and cbEGF32 domains is also flexible (Yuan et al., 2002). The TB6-cbEGF32 interface is less extensive than in other TB-cbEGF pairs, as two key packing motifs are missing (Jensen et al., 2005). Similarly, the EGF1 and EGF2 domains lack packing residues found in the FUN-EGF1 and EGF2-EGF3 pairs (an aromatic residue between C5 and C6 in EGF1, and the proline between C3 and C4 in EGF2), as well as packing residues in the interdomain linker. The flexibility of the linker means that the orientation of FUN-EGF1 relative to the rest of fibrillin is likely to be variable. There are few other candidates for flexible interdomain regions, although it has been speculated that the Pro-rich region could act as a hinge (Pereira et al., 1993). Our structure allows us to further improve the model for fibrillin organization (Figure 9A). Despite flexibility in the N-terminal region, the overall linear shape of fibrillin excludes the possibility of N-C-terminal interactions within the same molecule.

Structure-informed mutagenesis of FUN-EGF3 provided insights into the binding site for cbEGF41-43. A pull-down assay for the N-C interaction was established using the immobilized cbEGF41-43 fragment. Multimerization of the fibrillin C-terminal domains enhances their apparent affinity for binding to the fibrillin N-terminal region (Hubmacher et al., 2008). Thus, clustering of cbEGF41-43 (the minimal fragment that binds to the N-terminal domains) on beads is likely to mimic the in vivo situation. A comparison of the binding of FUN-EGF3 to cbEGF41-43 with smaller N-terminal fragments demonstrated that multiple domains contribute to the interaction. Furthermore, the EGF1-EGF2 linker was shown to play an important role. Flexibility of this region means that the two domain pairs could potentially fold back to interact with the C terminus (Figure 9B). In addition, the R62A substitution and deletion of the ^63^Y-N-A-Y^66^ motif, which are both in the flexible loop of the FUN domain, also resulted in reduced binding. Their involvement suggests which face of FUN-EGF1 contributes to the interaction (Figure 9C).

We also identified residues in FUN-EGF3 that contribute to heparin binding. Previous work showed that MFS-associated substitution T101A reduced the heparin-binding affinity of the longer FUN-EGF4 fragment (Cain et al., 2005). T101 is partially buried in the FUN-EGF3 structure, strongly suggesting that the substitution had an indirect effect by perturbing protein folding. Our results indicate that surface-accessible, positively charged residues, particularly R62, contribute to heparin binding. The overlap of the heparin and cbEGF41-43 binding sites suggests that HS may regulate the N-C-terminal interaction. Indeed, the addition of heparin or HS blocks microfibril assembly in cell culture, as does inhibition of HS synthesis (Tiedemann et al., 2001). A plausible explanation for these effects is that HS sequesters fibrillin monomers at the cell surface, preventing premature N-C interactions. Subsequent oligomerization of the C-terminal domains creates a high-affinity binding site for the N-terminal domains, which competes with the binding of FUN-EGF3 to HS. This results in regulated end-to-end assembly of fibrillin aggregates (Figure 9D).

The FUN-EGF3 structure will allow the binding sites of other elastic fiber molecules on fibrillin to be mapped in detail. The N164S residue substitution, which is associated with dominant ectopia lentis, perturbed binding of the FUN-EGF4 fragment to LTBP-4 (Ono et al., 2009). Identifying the location of this residue suggests which face of the EGF3 domain forms the binding site for LTBP-4 (Figure 9C).

In summary, we have determined the structure of the functionally important N-terminal domains of human fibrillin-1, which constitute the minimal interaction site for the fibrillin C terminus. As well as helping to identify regions involved in binding the C terminus and heparin, this structure will be essential for future studies of fibrillin interactions with elastic fiber molecules.

## Experimental Procedures

### Cloning and Site-Directed Mutagenesis

Sequences encoding the FUN-EGF3 and cbEGF41-43 regions of human fibrillin-1 were cloned into the pQE-30 vector (QIAGEN) for protein expression in *Escherichia coli* as described previously (Knott et al., 1996). A modified vector was used for constructs with C-terminal BirA tags (Cordle et al., 2008b). Site-directed mutagenesis was performed using the QuikChange Lightning mutagenesis kit (Agilent). The FUN-EGF3 (G5) mutant was cloned using an overlapping PCR.

### Protein Expression, Purification, Refolding, and Characterization

Protein expression, isotopic labeling, and purification protocols were similar to those described previously (Cordle et al., 2008b; Knott et al., 1996; Yadin et al., 2012). All proteins required the addition of 50% (v/v) glycerol to the refolding mixture. Refolding of cbEGF41-43 was carried out at pH 9.0 to maintain solubility. BirA-tagged proteins were biotinylated using an established method (Cordle et al., 2008b). SDS-PAGE analysis of protein fragments is shown in Figure S3. Heparin-binding experiments were performed using a HiTrap Heparin column (GE Healthcare) on an ÄKTA system. Proteins were diluted in buffer consisting of 20 mM Tris-HCl pH 7.4 and 150 mM NaCl before they were loaded onto the column.

### Pull-Down Protein-Protein Interaction Assay

Biotinylated cbEGF41-43 or the control protein cbEGF32-34 was immobilized on M270 streptavidin-coated magnetic beads (Life Technologies) in buffer comprising 50 mM Tris-HCl pH 9.5, 150 mM NaCl, 2 mM CaCl_2_, 0.05% (v/v) Tween-20, and 1% (w/v) BSA (Sigma-Aldrich). Washed beads were then incubated with 15 μg His-tagged protein in the same buffer, but at pH 7.4 instead of pH 9.5, for 1 hr. The beads were then washed with 50 mM Tris-HCl pH 7.4, 150 mM NaCl, 0.05% (v/v) Tween-20, and boiled in reducing SDS-PAGE sample buffer. Samples were analyzed by SDS-PAGE and western blotting. His-tagged protein was detected using an anti-RGS-His-horseradish peroxidase (HRP) conjugated antibody (QIAGEN) and biotinylated protein was detected using streptavidin-peroxidase (Sigma-Aldrich). Jagged-1 DSL-EGF3 control protein was kindly provided by Dr. Chandramouli Chillakuri.

### NMR Spectroscopy

The NMR experiments used for resonance assignment and data processing were described previously (Yadin et al., 2012). For the results described here, spectra were acquired using in-house-built spectrometers with Oxford Instrument magnets, GE/Omega consoles, and ^1^H operating frequencies of 600 or 750 MHz. NMR experiments for structure determination and dynamics studies were carried out using ^15^N- or ^13^C/^15^N-labeled FUN-EGF3 at a concentration of 1.5 mM at pH 5.4 and 298 K. Three-dimensional (3D) ^15^N-edited and ^13^C-edited NOE spectroscopy (NOESY)-heteronuclear single-quantum correlation spectroscopy (HSQC) spectra (mixing times 75 and 150 ms) were acquired at 750 MHz for FUN-EGF3 in 5% D_2_O/95% H_2_O (v/v) and 100% D_2_O, respectively. ^1^H-^15^N RDCs were measured using a bicelle alignment medium at 308 K. ^15^N relaxation experiments were performed at 600 MHz. Heteronuclear NOE ratios and longitudinal (T_1_) and transverse (T_2_) relaxation times were measured using established pulse sequences as described previously (Kay et al., 1989a).

### Structure Determination

Distance restraints for structure calculation were derived from 3D ^15^N-edited and ^13^C-edited NOESY spectra (mixing time 75 ms). Cross-peaks were assigned manually using published resonance assignments (Yadin et al., 2012). The ϕ angle restraints for some residues were obtained using ^3^J_HNHα_ values from a heteronuclear multiple-quantum correlation spectroscopy (HMQC)-J experiment (Kay et al., 1989b). Restraints of −120° ± 40° were used for residues with ^3^J_HNHα_ > 8 Hz. Additional ϕ and ψ torsion angle restraints were obtained using TALOS+ predictions on the basis of assigned chemical shifts (Shen et al., 2009). Simulated annealing and refinement calculations were performed using Xplor-NIH 2.29 (Schwieters et al., 2006). Structures were refined with a Rama torsion angle database potential (Kuszewski et al., 1996), a potential of mean force for hydrogen bond donor-acceptor pairs (Grishaev and Bax, 2004), and RDCs. The axial and rhombic components for the alignment tensors (separate values used for FUN-EGF1 and EGF2-EGF3) were calculated from partially refined structures using in-house-developed software. Ramachandran validation statistics were calculated using Procheck (Laskowski et al., 1996).

### Analysis of Dynamics Data

T_1_ and T_2_ values were obtained by fitting single exponential decays to peak intensities measured with 12 different delay times. Heteronuclear NOE ratios were calculated from the peak intensities of spectra with and without ^1^H presaturation. Errors were estimated using 500 Monte Carlo simulations, with the baseline noise as a measure of peak height error, as described previously (Bruylants and Redfield, 2009).

For further details regarding the materials and methods used in this work, see Supplemental Experimental Procedures.

## Figures and Tables

**Table 1:** NMR Structure Calculation Statistics

	FUN-EGF3 (R45–E178)	FUN-EGF1 (A53–C111)	EGF2-EGF3 (C119–E178)
NOE-derived distance restraints			
Total	2,599	1,559	1,040
Intraresidue	890	516	374
Interresidue	1,434	893	541
Sequential (| i-j | = 1)	571	340	231
Short-range (| i-j | < 5)	231	147	84
Long-range (| i-j | ≥ 5)	632	406	226
Ambiguous	275	150	125
Hydrogen bond restraints	30	18	12
Dihedral angle restraints			
Total	137	69	68
φ	73	39	34
ψ	64	30	34
RDCs			
^1^D_NH_	54	28	26
Total number of restraints	2,820	1,674	1,146
Restraint violations			
Distance restraint violations > 0.5 Å	0	0	0
Dihedral angle violations > 5°	0	0	0
Rmsd from experimental restraints			
Distance restraints (Å)	0.024 ± 0.001	0.025 ± 0.001	0.024 ± 0.003
Dihedral angle restraints (°)	0.338 ± 0.109	0.194 ± 0.141	0.408 ± 0.171
RDC restraints (Hz)	0.822 ± 0.074	0.755 ± 0.078	0.884 ± 0.110
Rmsd from idealized geometry			
Bonds (Å)	0.004 ± 0.000	0.004 ± 0.000	0.003 ± 0.000
Angles (°)	0.513 ± 0.016	0.552 ± 0.016	0.501 ± 0.019
Impropers (°)	0.365 ± 0.007	0.417 ± 0.016	0.337 ± 0.015
Ramachandran plot			
Residues in most favored regions (%)	80.5	81.8	81.9
Residues in additional allowed regions (%)	16.0	18.1	13.4
Residues in generously allowed regions (%)	1.6	0.1	1.7
Residues in disallowed regions (%)	1.9	0.0	3.0
Coordinate precision (rmsd; Å)			
Backbone	N/A	0.586 ± 0.196	1.166 ± 0.288
Heavy atom	N/A	0.916 ± 0.246	1.820 ± 0.305

Statistics are given for full-length FUN-EGF3 and separately for the two domain pairs. Quality statistics were calculated using Xplor-NIH. Restraint violations and rmsd values are given as mean values per structure ± the SD. Ramachandran statistics were calculated using Procheck (Laskowski et al., 1996). N/A, not applicable.

## Accession Numbers

The atomic coordinates of the FUN-EGF3 ensemble and structure calculation restraints have been deposited in the Protein Data Bank under accession number 2M74.

## Supplemental Information

Document S1. Supplemental Experimental Procedures, Figures S1–S3, and Tables S1–S3
