# Supplementary material for: Structure of the Fibrillin-1 N-Terminal Domains Suggests that Heparan Sulfate Regulates the Early Stages of Microfibril Assembly
Source: Structure. 2013 Oct 8;21(10):1743–56. doi: 10.1016/j.str.2013.08.004 (PMC3794157; doi:10.1016/j.str.2013.08.004)
Supplement: Document S1. Supplemental Experimental Procedures, Figures S1–S3, and Tables S1–S3 [file mmc1.pdf]

Structure, Volume *21*

Supplemental Information

Structure of the Fibrillin-1 N-Terminal Domains Suggests that Heparan Sulfate Regulates the Early Stages of Microfibril Assembly

David A. Yadin, Ian B. Robertson, Joanne McNaught-Davis, Paul Evans, David Stoddart, Penny A. Handford, Sacha A. Jensen, and Christina Redfield

Inventory of Supplemental Information

Figure S1. Streptavidin-HRP Western blots of pull-down assay samples. (related to Figure 1C, Figure 8A and 8D). These Western blots confirmed the presence of immobilised proteins in the pull-down assays shown in Figure 1C, 8A and 8D.

Figure S2. Effects of MFS mutations on the human fibrillin-1 N-terminus. (related to Figure 5D) This figure shows SDS-PAGE characterisation and trafficking data for three Marfan syndrome mutations affecting interdomain interfaces illustrated in Figure 5D.

Figure S3. Characterisation of proteins used in this study by gel electrophoresis and NMR spectroscopy. (related to Figure 8) This figure shows SDS-PAGE and NMR characterisation of the shorter fibrillin-1 fragments and mutants used in pull-down assays to characterise the interaction interface between FUN-EGF3 and EGF41-43.

Table S1. Heteronuclear NOE ratios for NH_2 side chains of glutamine and asparagine residues in FUN-EGF3 (related to Figure 3B). The elevated values of ~0.6 determined for N57 and N125 are consistent with the location of these residues in interdomain interfaces.

Table S2 Analysis of cross-peak markers in 2D ^{1}H homonuclear NOESY spectra of FUN-EGF3 mutants. (related to Figure 5D). These NOEs are used to characterise the effects of the Marfan syndrome mutations on the structure.

Table S3. Protein sequences of the FUN-EGF3 region from a range of species (related to Figure 7). These sequences were used to produce the alignments shown in Figure 7.

Supplemental Information

Structure of the fibrillin-1 N-terminal domains suggests heparan sulphate regulates the early stages of microfibril assembly

Supplemental Data

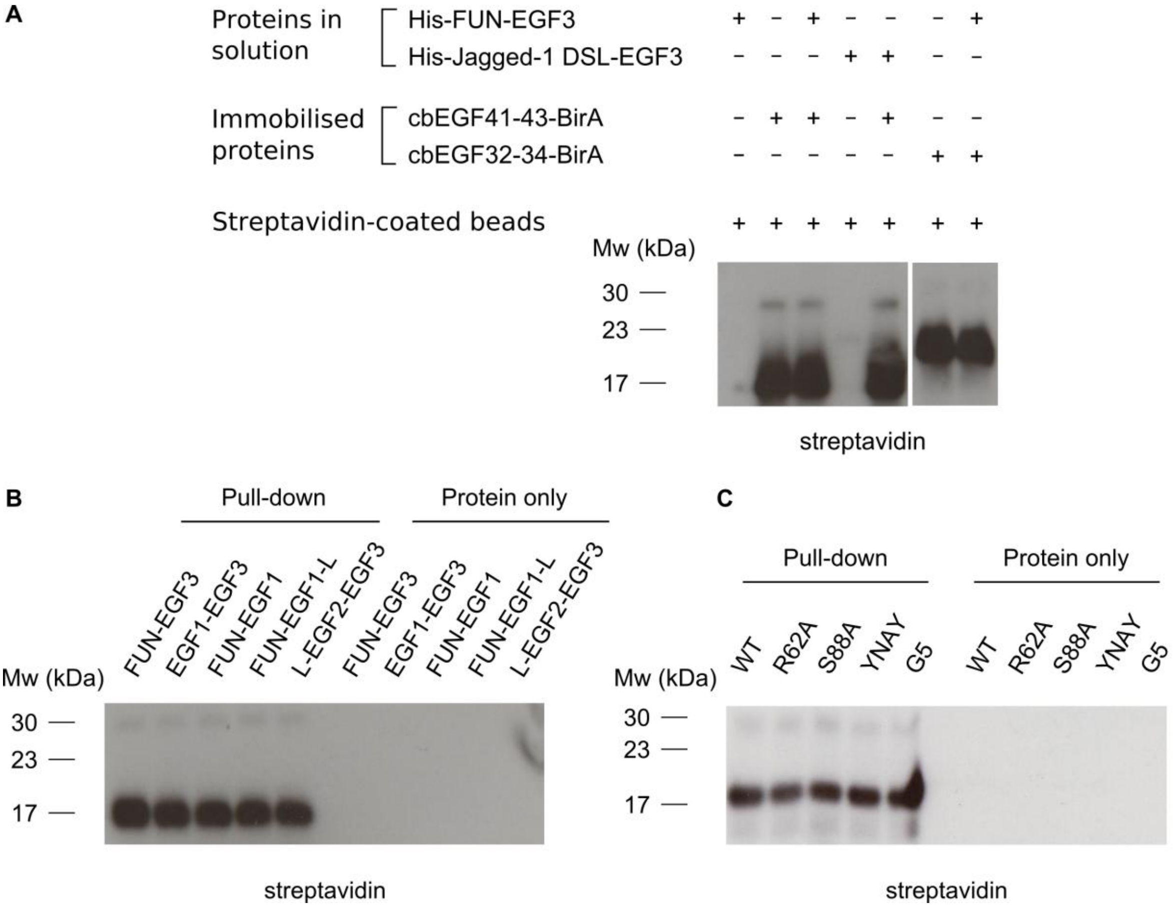

Figure S1. Streptavidin-HRP Western blots of pull-down assay samples.

A) A second Western blot was performed with samples from the FUN-EGF3-cbEGF41-43 pull-downs (anti-RGS-His blot shown in Figure 1C). Streptavidin-HRP was used to detect the site-specifically biotinylated cbEGF41-43-BirA and cbEGF32-34-BirA proteins. B) Streptavidin-HRP Western blot from fragment pull-downs (Figure 8A). C) Streptavidin-HRP Western blot from FUN-EGF3 mutant pull-downs (Figure 8D).

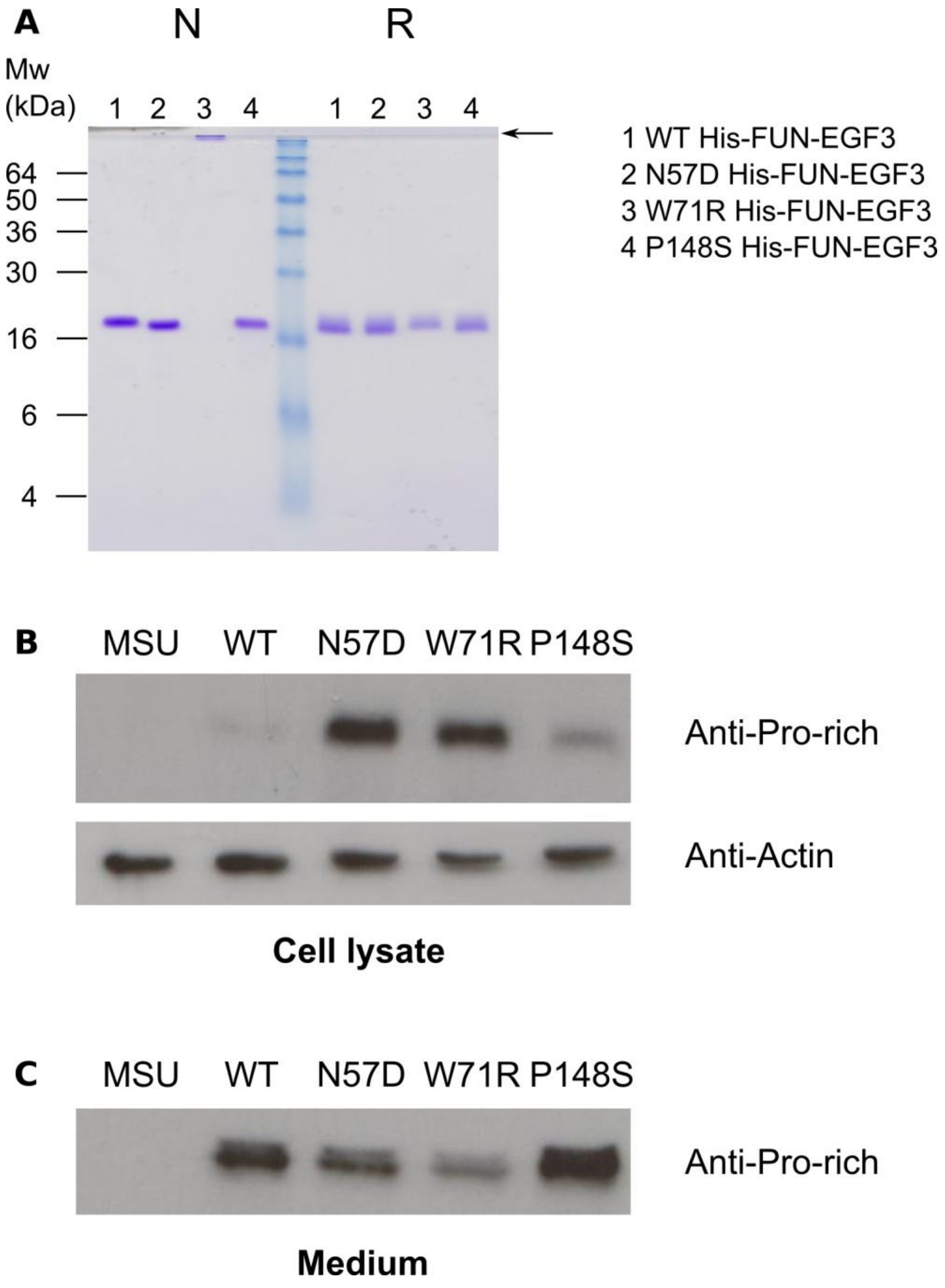

Figure S2. Effects of MFS mutations on the human fibrillin-1 N-terminus.

Figure S2 (cont). Effects of MFS mutations on the human fibrillin-1 N-terminus.

A) 16% SDS-PAGE gel of His-tagged FUN-EGF3 fragments with MFS-associated amino acid substitutions (Figure 5D). Samples were prepared in the absence (N) and presence (R) of the reducing agent 2-mercaptoethanol. The fragment with the W71R substitution runs as a high molecular weight band under non-reducing conditions, but as a monomer with the expected mobility under reducing conditions. This indicates that this fragment is misfolded. B) Western blots of cell lysate samples from untransfected (MSU) and MSU1.1 cells transfected with pKG-52 vectors encoding wildtype (WT) and mutant forms (N57D, W71R, P148S) of the fibrillin-1 Nterm-Pro fragment. Cells were cultured for three days after splitting before Western blot analysis. Top: blot with anti-Pro-rich antibody, showing the Nterm-Pro bands (~48 kDa); bottom: loading control blot with anti-α-actin antibody, showing the actin band running at a molecular weight of ~42 kDa. C) Western blot of conditioned medium samples using the anti-Pro-rich antibody, showing the Nterm-Pro bands in samples from transfected cells. The results show increased intracellular retention of the N57D and W71R mutant proteins compared to the WT, while the P148S substitution has no obvious effect.

Figure S3. Characterisation of proteins used in this study by gel electrophoresis and NMR spectroscopy.

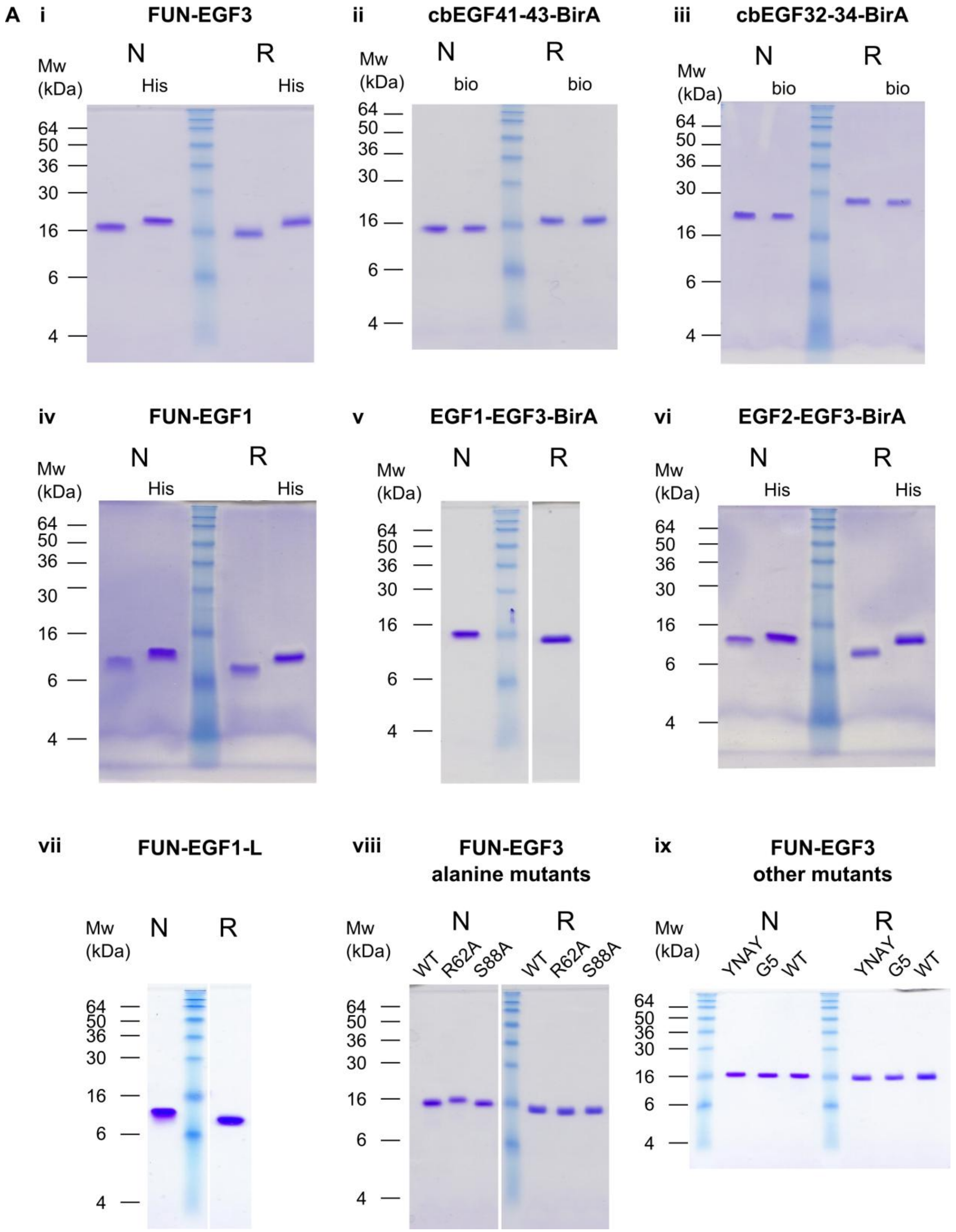

B

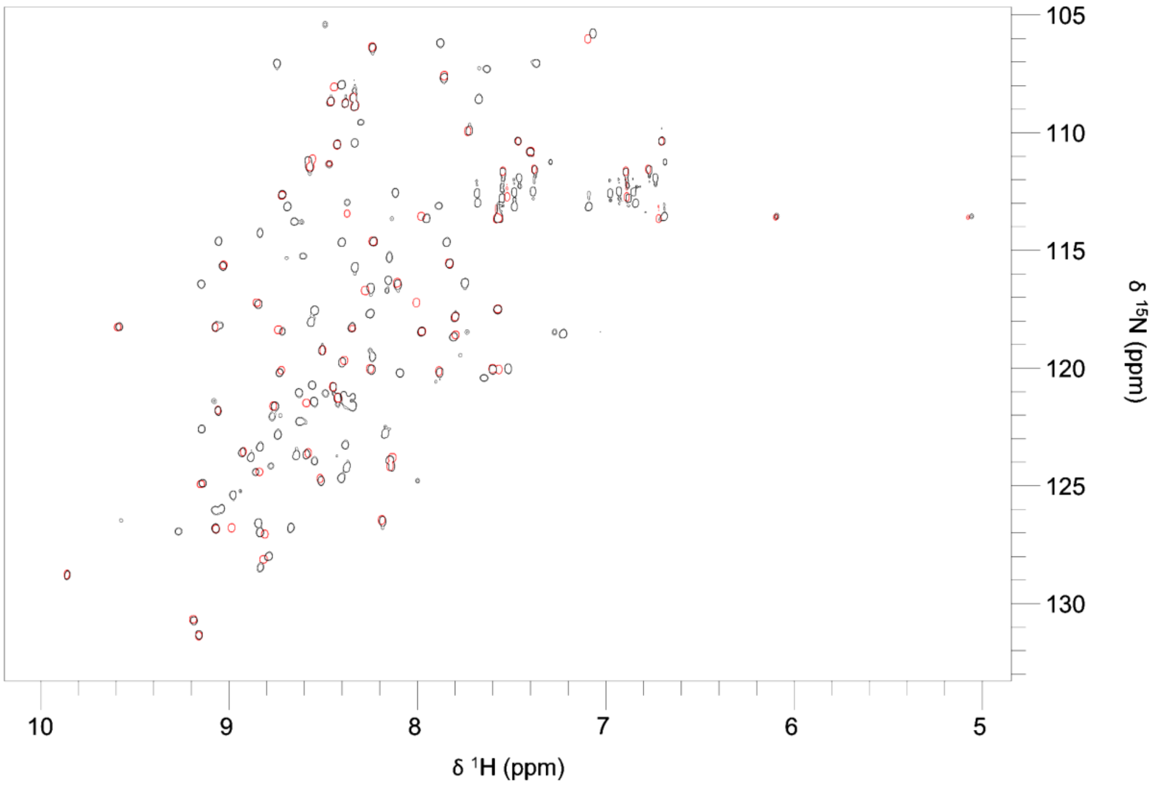

105
110
115
120
125
130
δ ¹⁵N (ppm)
10
9
8
7
6
5
δ ¹H (ppm)

C

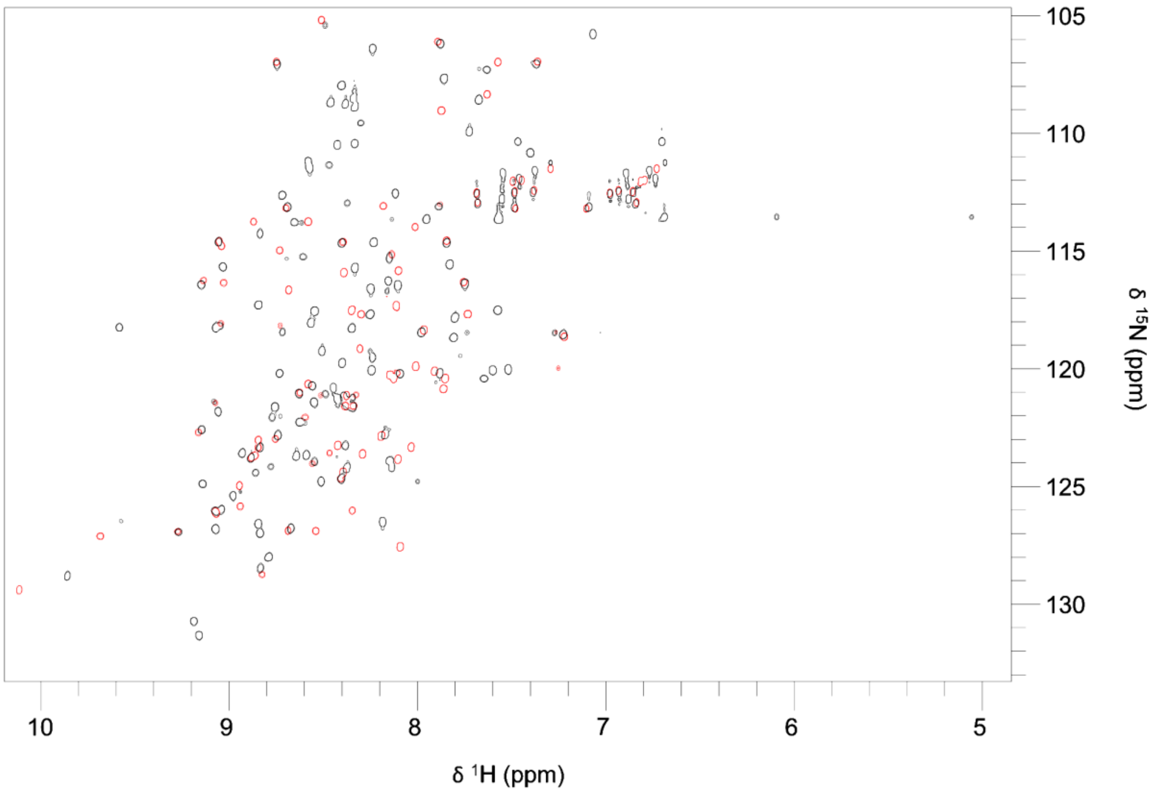

105
110
115
120
125
130
δ ¹⁵N (ppm)
10
9
8
7
6
5
δ ¹H (ppm)

D

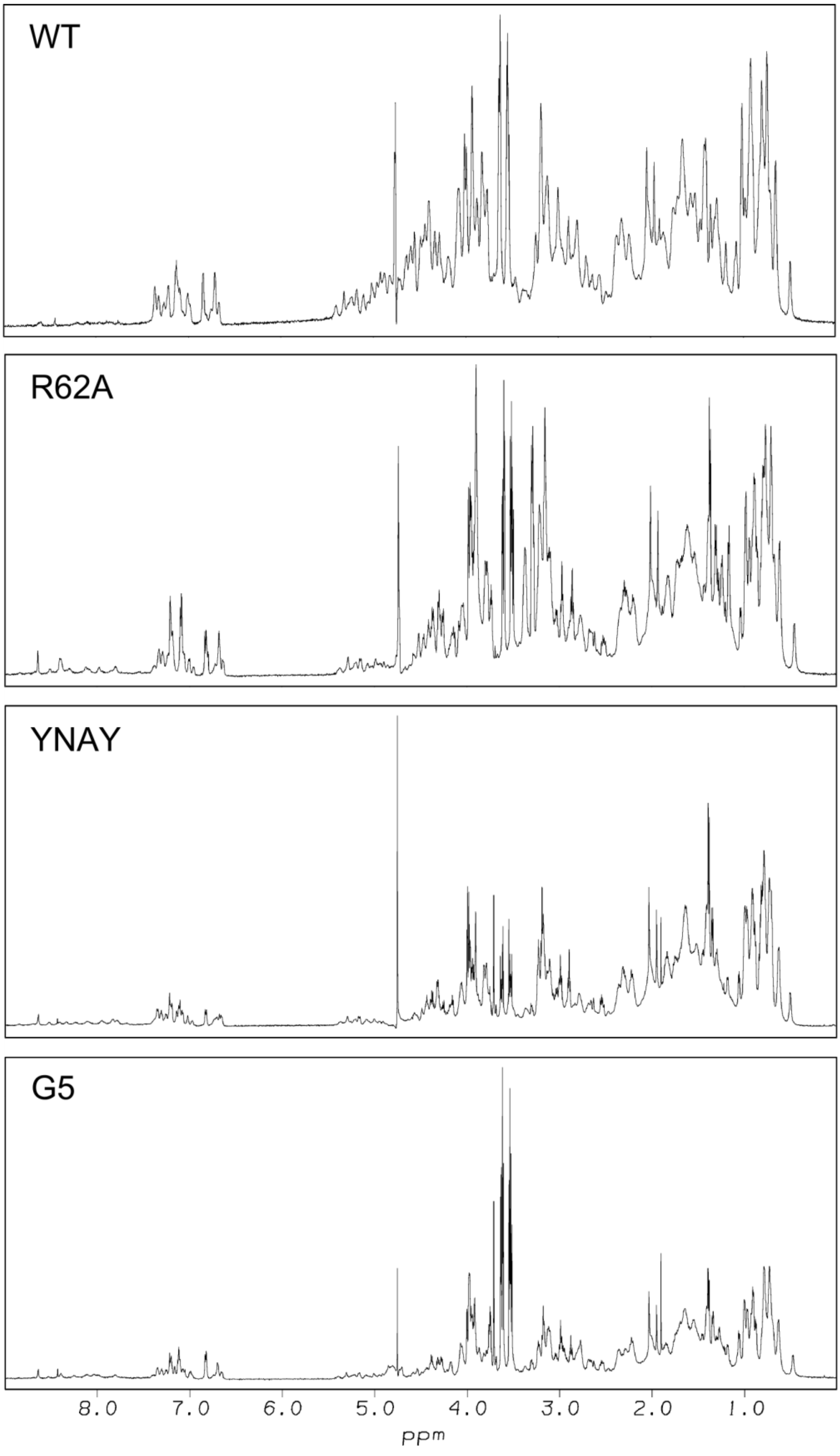

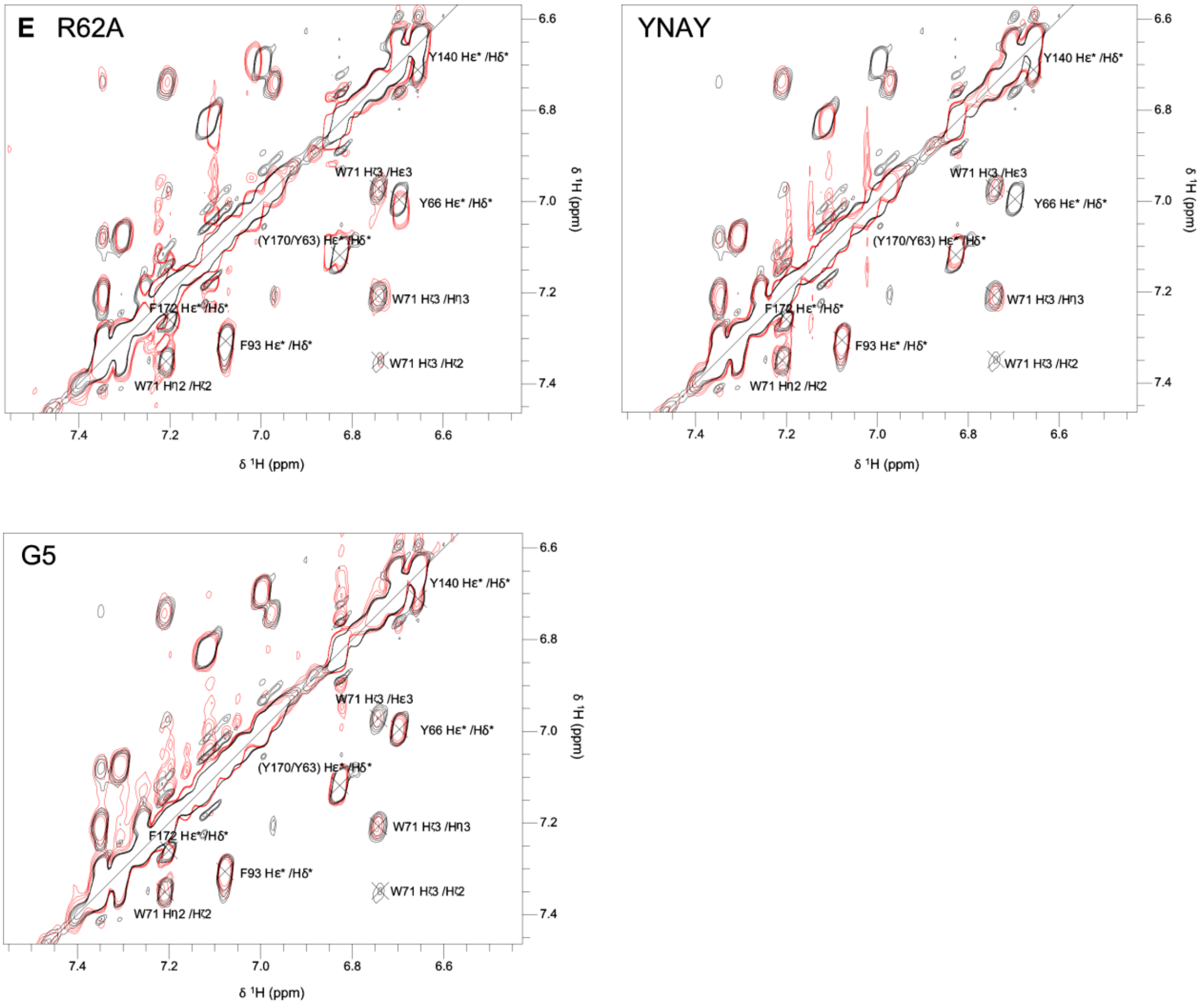

Figure S3 (cont). Characterisation of proteins used in this study by gel electrophoresis and NMR spectroscopy.

A) 16% SDS-PAGE gels of fibrillin fragments and mutants described in this study (Figure 8). “His” indicates that the protein is His-tagged, while “bio” indicates that a BirA-tagged protein is biotinylated. Samples were prepared in the absence (N) and presence (R) of the reducing agent 2-mercaptoethanol. i) FUN-EGF3: His-tagged and factor Xa-cleaved. ii) cbEGF41-43-BirA: His-tagged and factor Xa-cleaved. iii) cbEGF32-34-BirA: His-tagged and factor Xa-cleaved. iv) FUN-EGF1: His-tagged and factor Xa-cleaved. v) EGF1-EGF3-BirA: His-tagged. vi) EGF2-EGF3-BirA: His-tagged and factor Xa-cleaved. vii) FUN-EGF1-L: His-tagged. viii) FUN-EGF3 alanine mutants R62A and S88A alongside WT protein: factor Xa-cleaved. ix) FUN-EGF3 mutants YNAY and G5 alongside WT protein: factor Xa-cleaved.

Figure S3 (cont). Characterisation of proteins used in this study by gel electrophoresis and NMR spectroscopy.

NMR was used to compare the structures of fibrillin N-terminal fragments and FUN-EGF3 mutants with wildtype FUN-EGF3 (Figure 8). B) Two-dimensional (2D) ^{1}H-^{15}N HSQC spectra of ^{15}N-labelled FUN-EGF1 and FUN-EGF3, acquired in 5% D_2O/95% H_2O (v/v) at pH 5.4 and 298 K. Contours of FUN-EGF1 spectrum (red) are superimposed on the spectrum of FUN-EGF3 (black). Peaks in the FUN-EGF1 spectrum coincide with equivalent peaks assigned to residues in the FUN-EGF1 region of FUN-EGF3. C) Contours of HSQC spectrum of ^{15}N-labelled L-EGF2-EGF3 (red) superimposed on the spectrum of FUN-EGF3 (black). Peaks in the L-EGF2-EGF3 spectrum generally coincide with equivalent peaks from residues in the L-EGF2-EGF3 region of FUN-EGF3, but there are differences in residues from the EGF3 domain. This is due to a C-terminal BirA tag in the L-EGF2-EGF3 fragment. D) 1D ^{1}H spectra of WT, R62A, YNAY and G5 FUN-EGF3 proteins. Spectra were acquired of samples in 100% D_2O at pH 5.4 and 298 K. They indicate that the mutant proteins adopt a similar fold to the WT protein. Upfield-shifted methyl resonances, downfield-shifted Hα resonances and slowly exchanging amides are all markers for folded protein. E) More detailed analysis of folding of FUN-EGF3 mutants using 2D ^{1}H NOESY spectra. Spectra were acquired of samples in 100% D_2O at pH 5.4 and 298 K. The aromatic region of each mutant FUN-EGF3 spectrum (R62A, YNAY, G5; red contours) is superimposed on the WT FUN-EGF3 spectrum (black). The chemical shifts of aromatic side-chain resonances are good indicators of correct folding in FUN-EGF3: Y63 and Y66 are in the FUN domain, W71 is at the FUN-EGF1 interface, F93 is in the EGF1 domain, Y140 is at the EGF2-EGF3 interface, and Y170 and F172 are in the EGF3 domain. E) YNAY FUN-EGF3. The Y66 ^{1}Hε-^{1}Hδ cross-peak is missing from the YNAY spectrum, as expected from the deletion of the Y-N-A-Y motif.

Table S1

Residue	Peak 1		Peak 2	
	**NOE**	**Error**	**NOE**	**Error**
**N57**	0.646	0.072	0.645	0.059
**Q64**	-0.388	0.01	-0.376	0.01
**N78**	0.37	0.026	0.358	0.025
**Q79**	-0.481	0.012	-0.489	0.013
**N98**	0.17	0.024	–	–
**Q106**	-1.215	0.14	-1.003	0.01
**Q117**	-1.399	0.01	-1.435	0.011
**N120***	0	0	–	–
**N125**	0.627	0.128	0.669	0.085
**Q137**	-0.85	0.016	-0.871	0.015
**Q147**	-0.78	0.099	-0.725	0.077
**N164**	0.122	0.022	0.244	0.026
**Q176**	-0.59	0.016	-0.589	0.014

Heteronuclear NOE ratios for NH_2 side chains of glutamine and asparagine residues in FUN-EGF3 (Figure 3). These values were measured in a separate experiment from the backbone NOE ratios, as described previously (Buck et al., 1995). No value was calculated for the second cross-peak of N98 due to peak overlap. There were no obvious peaks for N120 in the spectrum with pre-saturation and so the values are listed as 0. The values of ~0.6 determined for N57 and N125 are consistent with the location of these residues in interdomain interfaces.

Table S2

	**WT**	**N57D**	**P148S**
**FUN domain**			
V58 H^{α}/C67 H^{α}	4.06, 5.23	*	4.06, 5.23
Y66 $H^{\varepsilon*}$/$H^{\delta*}$	6.70, 7.00	*	6.70, 7.00
T73 H^{β}/H^{α}	4.44, 5.30	*	
**FUN-EGF1 interface**			
W71 $H^{\zeta3}$/$H^{\eta2}$	6.74, 7.21	*	6.74, 7.21
W71 H^{α}/P83 H^{α}	4.17, 5.39	*	4.17, 5.38
**EGF1 domain**			
F93 $H^{\varepsilon*}$/$H^{\delta*}$	7.08, 7.31	7.08, 7.31	7.07, 7.30
N98 H^{α}/C85 H^{α}	4.46, 4.95	4.46, 4.96	4.45, 4.95
T101 H^{α}/I107 H^{α}	4.43, 5.17	4.42, 5.17	4.43, 5.17
**EGF2 domain**			
D132 H^{α}/$H^{\beta*}$	2.79, 4.37	2.79, 4.37	2.79, 4.37
**EGF2-EGF3 interface**			
Y140 $H^{\varepsilon*}$/$H^{\delta*}$	6.66, 6.71	6.66, 6.71	6.68, 6.81**
Y140 H^{α}/P148 H^{α}	4.72, 5.26	Unclear	*
V149 $H^{\gamma*}$/H^{α}	0.74, 3.94	0.74, 3.94	*
**EGF3 domain**			
C160 H^{α}/C166 H^{α}	4.87, 5.31	4.87, 5.31	*
N164 H^{α}/C150 H^{α}	4.47, 5.10	4.47, 5.10	4.48, 5.09

Analysis of cross-peak markers in 2D ^{1}H homonuclear NOESY spectra of FUN-EGF3 mutants (Figure 5D). Marker peaks found in the spectrum of wildtype (WT) FUN-EGF3 are listed according to their location in the structure. Chemical shift values for these cross-peaks are given. For the mutants (N57D and W71R), * indicates that a peak was missing. Peaks with a combined chemical shift difference compared to the WT of greater than 0.03 ppm are marked **. The spectrum of N57D FUN-EGF3 lacked peaks associated with the FUN domain and FUN-EGF1 interface, while peaks associated with the EGF2-EGF3 interface and EGF3 domain were different in P148S FUN-EGF3. These differences indicated perturbations to the folding of these mutants.

Organism	Accession number	Database
Homo sapiens (human)	NP_000129.3 (fibrillin-1)	NCBI
	NP_001990.2 (fibrillin-2)	NCBI
	NP_115823.3 (fibrillin-3)	NCBI
	NP_996826.2 (LTBP-1L)	NCBI
Canis familiaris (dog)	XP_535468.2 (fibrillin-1)	NCBI
Mus musculus (mouse)	NP_032019.2 (fibrillin-1)	NCBI
Gallus gallus (chicken)	XP_413815.3 (fibrillin-1)	NCBI
Xenopus tropicalis (clawed frog)	XP_002936615.1	NCBI
Danio rerio (zebrafish)	NP_001129262.1 (fibrillin-2)	NCBI
Petromyzon marinus (lamprey)	ENSPMAP00000002691	Ensembl
Ciona intestinalis (sea squirt)	XP_002119988.1	NCBI
Tribolium castaneum (red flour beetle)	XP_974344.2	NCBI
Nematostella vectensis (starlet sea anenome)	A7SBY7_NEMVE (N-terminal fragment)	Ensembl

Table S3. Protein sequences were obtained from the National Center for Biotechnology Information (NCBI; http://www.ncbi.nlm.nih.gov/) and Ensembl databases (http://www.ensembl.org/index.html) (Figure 7).

Supplemental Experimental Procedures

Measurement of residual dipolar couplings (RDCs)

Sample conditions for RDC measurement were 0.5 mM protein with 5% (w/v) bicelles comprising the ether linked lipids 1,2-O-ditridecyl-*sn*-glycero-3-phosphocholine and 1,2-dihexyl-*sn*-glycero-3-phospho-choline (Avanti Polar Lipids), as well as cetyl trimethyl ammonium bromide (Sigma-Aldrich) in a molar ratio of 35:10:1. RDCs were calculated from peak positions in ^{1}H-^{15}N IPAP-HSQC spectra (Ottiger et al., 1998) acquired at 308 K for isotropic and aligned bicelle solutions. Assignments at 308 K were obtained from a series of 2D ^{1}H-^{15}N HSQC spectra acquired at 298, 303 and 308 K.

Buried surface area and twist/tilt calculations

Buried surface areas were calculated using Areaimol, part of the CCP4 software package (Collaborative Computational Project, 1994). Accessible surface areas were calculated separately for the complete domain pair (FUN-EGF1 or EGF2-EGF3) and two domains of the pair. The buried surface for each domain was calculated as the difference between the accessible surface area in the presence and absence of the opposing domain of the pair. The values for the two halves were then averaged.

Twist and tilt angle calculations were performed using Mod2 software (Downing et al., 1996). For the fibrillin EGF2-EGF3 pair, the planes of each domain were defined using the β-hairpin structures: G127-S130 and H133-C136 for EGF2; G158-V161 and R165-C168 for EGF3.

Protein secretion assay

The Nterm-Pro fibrillin fragment has been previously used for protein secretion studies in MSU1.1 human fibroblast cells (Kettle et al., 2000; Whiteman and Handford, 2003). pKG-52 vector DNA encoding the N-term-Pro fragment was transfected into MSU1.1 cells − 24 µg per 75 cm^2 flask with 60 µl Lipofectamine-2000 (Life Technologies) added. Cells were incubated overnight in Opti-MEM medium with the DNA-lipofectamine mixture. The medium was then replaced with complete

Dulbecco's Modified Eagle Medium (DMEM). After two-three days cells were split 1:2 and re-seeded into DMEM containing 2.5 µg/ml puromycin (Sigma-Aldrich). Selection medium was changed twice-weekly for three weeks until ~50 colonies of ~3 mm diameter were observed. Clones were pooled for the secretion assay by splitting 1:4 into 35 cm^2 flasks. Cell lysate and medium samples were collected and analysed by Western blotting. The Nterm-Pro fragment was detected using Western blotting with the previously described antibody raised against the Pro-rich region of human fibrillin-1 (Kettle et al., 1999). Blotting with an α-actin antibody (A2547, Sigma-Aldrich) was used as a control for cell lysate samples.

Supplemental References

Buck, M., Boyd, J., Redfield, C., Mackenzie, D., Jeenes, D., Archer, D., and Dobson, C. (1995). Structural determinations of protein dynamics - analysis of N-15 NMR relaxation measurements for main-chain and side-chain nuclei of hen egg-white lysozyme. Biochemistry *34*, 4041-4055.

Collaborative Computational Project, N.m. (1994). The CCP4 suite: programs for protein crystallography. Acta Crystallogr D Biol Crystallogr *50*, 760-763.

Downing, A.K., Knott, V., Werner, J.M., Cardy, C.M., Campbell, I.D., and Handford, P.A. (1996). Solution structure of a pair of calcium-binding epidermal growth factor-like domains: implications for the Marfan syndrome and other genetic disorders. Cell *85*, 597.

Kettle, S., Cardy, C.M., Hutchinson, S., Sykes, B., and Handford, P.A. (2000). Characterisation of fibrillin-1 cDNA clones in a human fibroblast cell line that assembles microfibrils. Int J Biochem Cell Biol *32*, 201.

Kettle, S., Yuan, X., Grundy, G., Knott, V., Downing, A.K., and Handford, P.A. (1999). Defective calcium binding to fibrillin-1: consequence of an N2144S change for fibrillin-1 structure and function. J Mol Biol *285*, 1277-1287.

Ottiger, M., Delaglio, F., and Bax, A. (1998). Measurement of J and dipolar couplings from simplified two-dimensional NMR spectra. J Magn Reson *131*, 373-378.

Whiteman, P., and Handford, P.A. (2003). Defective secretion of recombinant fragments of fibrillin-1: implications of protein misfolding for the pathogenesis of Marfan syndrome and related disorders. Hum Mol Genet *12*, 727-737.
